# A conformational landscape for alginate secretion across the outer membrane of *Pseudomonas aeruginosa*

**DOI:** 10.1107/S1399004714001850

**Published:** 2014-07-25

**Authors:** Jingquan Tan, Sarah L. Rouse, Dianfan Li, Valerie E. Pye, Lutz Vogeley, Alette R. Brinth, Toufic El Arnaout, John C. Whitney, P. Lynne Howell, Mark S. P. Sansom, Martin Caffrey

**Affiliations:** a Schools of Medicine and Biochemistry and Immunology, Trinity College, Dublin, Ireland; b Department of Biochemistry, University of Oxford, South Parks Road, Oxford, England; c Program in Molecular Structure and Function, The Hospital for Sick Children, Toronto, Ontario, Canada; d University of Toronto, Toronto, Ontario, Canada

**Keywords:** alginate secretion, biofilm, lipidic cubic phase, membrane protein, molecular-dynamics simulation, porin

## Abstract

The exopolysaccharide alginate is an important component of biofilms produced by *Pseudomonas aeruginosa*, a major pathogen that contributes to the demise of cystic fibrosis patients. Alginate exits the cell *via* the outer membrane porin AlgE. X-ray structures of several AlgE crystal forms are reported here. Whilst all share a common β-barrel constitution, they differ in the degree to which loops L2 and T8 are ordered. L2 and T8 have been identified as an extracellular gate (E-gate) and a periplasmic gate (P-gate), respectively, that reside on either side of an alginate-selectivity pore located midway through AlgE. Passage of alginate across the membrane is proposed to be regulated by the sequential opening and closing of the two gates. In one crystal form, the selectivity pore contains a bound citrate. Because citrate mimics the uronate monomers of alginate, its location is taken to highlight a route through AlgE taken by alginate as it crosses the pore. Docking and molecular-dynamics simulations support and extend the proposed transport mechanism. Specifically, the P-gate and E-gate are flexible and move between open and closed states. Citrate can leave the selectivity pore bidirectionally. Alginate docks stably in a linear conformation through the open pore. To translate across the pore, a force is required that presumably is provided by the alginate-synthesis machinery. Accessing the open pore is facilitated by complex formation between AlgE and the periplasmic protein AlgK. Alginate can thread through a continuous pore in the complex, suggesting that AlgK pre-orients newly synthesized exopolysaccharide for delivery to AlgE.

## Abstract 2

Crystal structures of the β-barrel porin AlgE reveal a mechanism whereby alginate is exported from *P. aeruginosa* for biofilm formation.

## 1. Introduction   

*Pseudomonas aeruginosa* is an opportunistic pathogen that causes morbidity and mortality in patients with compromised immunity, such as cystic fibrosis sufferers and burns patients (Li *et al.*, 2005 ▶). At particular risk of disease are patients requiring extensive stays in intensive care units and cancer sufferers. *P. aeruginosa* is responsible for ∼10% of all hospital-acquired infections and is difficult to treat because of its naturally high antibiotic resistance. Resistance arises from low cell-envelope permeability, efflux pumps and biofilm formation (Hancock & Speert, 2000 ▶).

Virulence by *P. aeruginosa* involves the production of disease-causing secondary metabolites, nutrient scavenging, motility and biofilms. Biofilm formation occurs when the organism transitions from a planktonic or motile state to a matrix-embedded non-motile phenotype. The matrix of the biofilm, comprised of exopolysaccharides, proteins and DNA, provides structural stability and confers resistance to antibiotics and to the immune defences of the host (Høiby *et al.*, 2010 ▶; Grant & Hung, 2013 ▶).

The exopolysaccharide alginate is a component of so-called ‘mucoid’ biofilms produced by *P. aeruginosa*, making it an important virulence factor in its own right. Alginate is a random linear polymer of mannuronic acid (M) and guluronic acid (G). The M units, which derive from fructose 6-phosphate, are synthesized in the cytoplasm and are proposed to be polymerized and transported into the periplasm by the combined action of the integral membrane proteins Alg8 and Alg44 (Oglesby *et al.*, 2008 ▶; Rehman *et al.*, 2013 ▶). In the periplasm, M is randomly epimerized to G or partially acetylated, and the mature alginate is directed to the transmembrane β-barrel protein AlgE in the outer membrane for conveyance to the extracellular space. At least 13 proteins are involved in the synthesis of alginate and its movement across the cell envelope. 12 of these are encoded on the tightly regulated 17 kb *algD* operon (Ohman & Chakrabarty, 1981 ▶; Ohman *et al.*, 1985 ▶).

We have been working on membrane proteins involved in quorum sensing and virulence-factor production in *P. aeruginosa*, and AlgE emerged early on as a target of interest. Our objective was to obtain a high-resolution crystal structure of the protein with a view to understanding how it functions, as its mechanism was expected to differ from other capsular polysaccharide-export systems such as Wza (Dong *et al.*, 2006 ▶). During the course of our work, a crystal structure of AlgE (PDB entry 3rbh) was reported at a resolution of 2.3 Å (Whitney *et al.*, 2011 ▶). This structure was obtained using protein that had been expressed as inclusion bodies and subsequently refolded and crystallized in surfactant micelles by the *in surfo* method (Caffrey, 2003 ▶). Our approach to structure determination was different; it involved working with a presumably natively folded form of AlgE and crystallization using the lipid bilayer-based mesophase (*in meso*) method (Caffrey *et al.*, 2012 ▶). Here, we report the structure of AlgE obtained using the latter approach. A number of different crystal forms were obtained, leading to several structures, the best having a resolution of 1.9 Å. The structures differ in important ways from one another and from that of the original 3rbh model. Combined, they provide an approximate map of the conformational landscape underlying alginate transport. This forms the basis of a proposed mechanism for alginate transport involving AlgE. The mechanism is supported and extended by docking and multiscale molecular-dynamics simulations.

## 2. Materials and methods   

### 2.1. Gene construction, protein production and purification   

The full-length DNA sequence for the *algE* gene was amplified by PCR using the primers 5′-CACC**ATG**AACAGCTCCCGTTCCG-3′ and 5′-**TCA**GAAGCGCCAGATGAAGT-3′, with the start and stop codons shown in bold in the forward and reverse primers, respectively. The amplified gene was cloned into the pET200/D-TOPO vector using the TOPO Cloning Kit (Invitrogen) and was confirmed by sequencing (Eurofins MWG). The AlgE-pET200/D-TOPO construct was transformed into *Escherichia coli* BL21(DE3) Star chemically competent cells (Invitrogen). A seeding culture was prepared by inoculating 50 ml Luria–Bertani (LB) medium with a single colony from the transformed cells and growing the cell culture in a shaking incubator (Infors HT Multitron Standard) at 37°C and 180 rev min^−1^ for 18 h. The seeding culture was transferred and diluted 100 times into 4 × 1 l fresh LB medium and grown in a shaking incubator (Infors) at 37°C and 220 rev min^−1^. The optical density at 600 nm (OD_600_) was monitored with a Nanodrop spectrophotometer (Nanodrop 1000, Thermo Scientific) and the cell culture was cooled to and held at 4°C for 30 min after the OD_600_ reached 0.6 (typically 2.5 h). IPTG was added to the cell culture at 4°C to a final concentration of 1 m*M* to induce recombinant protein production. Growth was allowed to continue for a further 18 h in a shaking incubator at 180 rev min^−1^ and 18°C. Biomass was harvested by centrifugation at 3000*g* and 4°C for 20 min. Cells dispersed in 0.1 l 50 m*M* Tris–HCl pH 7.2 were broken by passing them three times through a cell disruptor (Emulsiflex C5, Avestin) at 103 MPa and 4°C. The suspension was centrifuged at 4000*g* and 4°C for 20 min to pellet intact cells and debris. The supernatant, containing both soluble proteins and membranes, was centrifuged at 100 000*g* and 4°C for 30 min. The pelleted membrane fraction, containing the recombinant AlgE, was solubilized in 0.1 l 1.5%(*w*/*v*) *n*-octyl-β-d-glucopyranoside (β-­OG; catalogue No. 0311, Affymetrix), 0.15 *M* KCl, 50 m*M* Tris–HCl pH 7.2. The suspension was stirred at 4°C for 2 h and centrifuged at 100 000*g* and 4°C for 30 min. The supernatant, containing solubilized AlgE, was collected and passed through a 0.45 µm filter to remove particulates (Filtropur S; catalogue No. 83.1826, Sarstedt). The filtrate was loaded onto a Sepharose-IMAC column (catalogue No. 17-0920-06, GE Healthcare) containing 20 ml of resin that had been pre-charged with 0.1 m*M* CuCl_2_ in water and pre-equilibrated in 50 m*M* Tris–HCl pH 7.2, 0.1%(*v*/*v*) *N*,*N*-dimethyldodecylamine *N*-oxide solution (LDAO; catalogue No. 40231, Sigma; buffer *A*) at 4°C using an ÄKTA FPLC (GE Healthcare). Detergent exchange from β-OG to LDAO was performed by passing 20 column volumes of buffer *A* through the Cu^2+^–IMAC column to which the protein was bound. AlgE was eluted with a gradient of 0–0.3 *M* ammonium chloride in buffer *A* (Rehm *et al.*, 1994 ▶). Fractions eluting between 80 and 120 m*M* ammonium chloride were collected and concentrated in a YM-50 Centricon (Millipore) before being loaded onto a Superdex 200 HiLoad 16/60 column (catalogue No. 28-9893-35, GE Healthcare) at 4°C on an ÄKTA FPLC. Gel filtration was carried out in 0.1 *M* NaCl, 50 m*M* Tris–HCl pH 7.2, 0.1%(*v*/*v*) LDAO (buffer *B*). The absorbance at 280 nm (*A*_280_) of the eluent was monitored and peak fractions were collected and concentrated to either 10 or 20 mg ml^−1^, depending on the type of crystallization end use, in a 50 kDa MWCO Centricon (catalogue No. UFC805024; Millipore). Protein purity was determined by SDS–PAGE on 12%(*w*/*v*) SDS Precast Gels (catalogue No. NXG01212; Expedeon) in a loading series that included 0.1, 1, 10 and 100 µg protein that had been boiled for 5 min in SDS sample buffer containing 2.5%(*w*/*v*) SDS, 0.002%(*w*/*v*) bromophenol blue, 10%(*v*/*v*) glycerol, 60 m*M* Tris–HCl pH 6.8. SDS–PAGE was carried out at 100 V for 2 h in a 4°C ice–water bath. Bands were visualized using Gelcode Bluesafe Protein Stain (catalogue No. 24594; Pierce). Images of stained gels were recorded using FluorChem SP (Alpha Innotech). The heat-modifiability of the AlgE protein was verified on SDS–PAGE by incubating the protein at 10 mg ml^−1^ in SDS sample buffer at 50°C for 0, 5, 20 and 120 min before running the electrophoresis in an ice–water mixture at 100 V for 2 h.

The yield of pure AlgE protein ranged from 1 to 2 mg per litre of cell culture. The protein concentration was assayed by measuring *A*_280_ using a Nanodrop spectrophotometer (molar extinction coefficient = 104 850 *M* ^−1^ cm^−1^ (Gasteiger *et al.*, 2005 ▶). The protein was stored at −80°C in 10 µl aliquots at 10–20 mg ml^−1^ in buffer *B* in preparation for biophysical characterization and crystallization. Once thawed, the protein was used directly for biophysical characterization and/or crystallization.

### 2.2. Spectroscopic analysis   

All spectrophotometric measurements were carried out at 20–22°C using AlgE protein samples solubilized in buffer *B*. Spectra were baseline-corrected using protein-free buffers. The average of three consecutive spectra recorded using aliquots from the same solution is reported. UV–visible spectroscopic analysis was performed using protein at 0.5 mg ml^−1^ in a 1 cm path-length quartz cuvette (Sigma–Aldrich, St Louis, Missouri, USA) with a UVIKONXL spectrophotometer (Northstar Scientific, Leeds, England). Spectra were recorded from 400 to 250 nm at a scanning speed of 200 nm min^−1^.

Fluorescence measurements were carried out using 0.1 mg ml^−1^ protein in a 3 mm path-length quartz cuvette (Hellma, Jena, Germany) with a FluoroMax-3 spectrofluorometer (Horiba, Kyoto, Japan). Emission spectra were recorded from 470 to 300 nm at 10 nm s^−1^ with an excitation wavelength of 280 nm and a slit corresponding to a spectral width of 2 nm.

Circular-dichroism (CD) analysis was carried out using 1.0 mg ml^−1^ protein in a 0.1 mm path-length quartz cuvette (Starna, Hainault, England) with a Jasco J-815 spectrometer (Jasco, Easton, Maryland, USA) at 20°C. Spectra from 320 to 180 nm were recorded at 20 nm min^−1^ in 1 nm steps with a bandwidth setting of 1 nm. Spectra were smoothened using the binomial function included in the Jasco spectra-analysis software package (v.1.54.03). The *DichroWeb* web server (Whitmore & Wallace, 2008 ▶) was used to calculate secondary-structure content using the *CDSSTR* algorithm (Sreerama & Woody, 2000 ▶) and the SMP180 reference set (Abdul-Gader *et al.*, 2011 ▶).

### 2.3. Crystallization and structure determination   

An initial crystallization trial was carried out at 20°C using monoolein (9.9 MAG; catalogue No. M239, Nu-Chek) and a protein solution at 20 mg ml^−1^ following a published protocol (Caffrey & Cherezov, 2009 ▶; Caffrey & Porter, 2010 ▶).

The lipidic cubic phase was made by mixing protein solution with monoolein in a 2:3 ratio by volume. 11 commercial crystallization screens were used, as follows: Crystal Screen HT (Hampton Research), Grid Screen (Hampton Research), Index HT (Hampton Research), MembFac HT (Hampton Research), SaltRX (Hampton Research), MemStar MemSys HT96 (Molecular Dimensions), PACT premier (Molecular Dimensions), MemGold (Molecular Dimensions), JBScreen (Jena Biosciences), JBScreen Membrane (Jena Biosciences) and Wizard I and II (Emerald Bio). Trials were set up using the SIAS *in meso* robot (Cherezov *et al.*, 2004 ▶) to dispense 50 nl protein-laden mesophase and 800 nl precipitant solution per well into glass sandwich plates. Out of 1056 conditions, six crystal hits were found. Thin needle-shaped crystals measuring 30 × ∼3 µm grew in 7–14 d and were harvested and snap-cooled in liquid nitrogen without added cryoprotectant following a published protocol (Li *et al.*, 2012 ▶). X-ray diffraction measurements carried out on the General Medicine and Cancer Institutes Collaborative Access Team (GM/CA-CAT) 23-ID-B beamline (Fischetti *et al.*, 2009 ▶; 20 × 20 µm beam size; 12 keV) at the Advanced Photon Source (APS; Argonne National Laboratory, Argonne, Illinois, USA) demonstrated that the crystals were proteinaceous. After rounds of optimization based on the initial precipitant conditions, protein concentration, additives (catalogue No. HR2-428, Hampton Research) and temperature (4, 16 and 20°C), thin plate-shaped crystals that grew to a maximum size of 75 × 20 × ∼3 µm at 20 °C within 14 d were obtained. The best crystals diffracted to a resolution of 2.8 Å.

To further optimize crystallization, several short-chain monoacylglycerols (MAGs) were synthesized (Coleman *et al.*, 2004 ▶; Caffrey *et al.*, 2009 ▶) and used as host lipids (Li *et al.*, 2011 ▶). These included 7.7 MAG, 7.8 MAG, 7.9 MAG, 8.8 MAG and 8.9 MAG. A protein solution at 10 mg ml^−1^ was used for reconstitution. For 7.7 MAG and 7.8 MAG, equal volumes of protein solution and lipid were combined to make the lipidic mesophase. For 7.9 MAG, 8.8 MAG and 8.9 MAG, the protein solution to lipid volume ratio was the same as that for monoolein. Crystallization trials were set up as described above. Crystals were harvested and tested for X-ray diffraction either on the 23-ID-B/D beamlines of the GM/CA-CAT (Fischetti *et al.*, 2009 ▶) at APS or on beamline I24 (Evans *et al.*, 2011 ▶; 10 × 10 µm beam size, 12.8 keV) at the Diamond Light Source (DLS, Didcot, England). Crystals grown in 7.8 MAG diffracted best (2.8 Å resolution), and this hosting lipid was chosen for further optimization. After optimizing around the initial hit conditions with 7.8 MAG, large crystals appeared after 14 d at 20°C in two different precipitants: (i) 18%(*v*/*v*) PEG 400, 0.1 *M* sodium citrate, 0.1 *M* Tris–HCl pH 7.5 and (ii) 1.0 *M* sodium acetate, 0.1 *M* sodium cacodylate pH 6.5. Crystals were harvested and three data sets were collected at GM/CA-CAT. The best crystal diffracted to 1.9 Å resolution and data were collected using a single, thin plate-shaped crystal grown in crystallization condition (i) to a maximum size of 150 × 150 µm. Data were indexed, scaled and merged using *iMosflm* (Leslie & Powell, 2007 ▶) and *SCALA* (Evans, 2006 ▶), revealing three crystal forms. Table 1 ▶ provides a summary of the data-collection and processing statistics.

Prior to the publication of PDB entry 3rbh, initial attempts to solve the AlgE structure by molecular replacement (MR) using available β-barrel structures and wide-search MR (Stokes-Rees & Sliz, 2010 ▶) against every known protein fold and *BALBES* (Long *et al.*, 2008 ▶) all failed to provide a solution. *Ab initio* phasing had been initiated but was halted when the *in surfo* structure of AlgE became available (Whitney *et al.*, 2011 ▶). Structures were solved by MR using *Phaser* (McCoy *et al.*, 2007 ▶) and the *in surfo* AlgE structure 3rbh (chain *A* with loops and bulky side chains removed) as a search model. Structures were refined by iterative cycles of (i) manual rebuilding in *Coot* (Emsley *et al.*, 2010 ▶) and (ii) reciprocal-space refinement using *PHENIX* (Adams *et al.*, 2010 ▶). Solvent and lipid molecules were fitted into positive *F*_o_ − *F*_c_ electron density where appropriate. The final structures were assessed using *MolProbity* (Chen *et al.*, 2010 ▶) before deposition into the Protein Data Bank in Europe (PDBe; http://www.ebi.ac.uk/pdbe/) with accession codes 4afk (AlgE-1.9), 4azl (AlgE-2.8) and 4b61 (AlgE-2.4). Structure analysis was carried out with *Coot* and the *CCP*4 (Winn *et al.*, 2011 ▶) toolset. Figures were prepared using *PyMOL* (Schrödinger). Refinement and model statistics are given in Table 1 ▶.

Attempts to obtain a structure of AlgE with di-mann­uronate bound were made by co-crystallization and soaking. Co-crystallization trials were set up with AlgE in 7.8 MAG at 20°C as described above, using a precipitant consisting of 0.9 *M* sodium acetate, 0.1 *M* sodium cacodylate pH 6.5 to which 0.01, 0.05 or 5 m*M* di-mannuronate was added. Di-mannuronate was produced as described in Whitney *et al.* (2011 ▶). Most conditions produced crystals and a complete data set to 3.0 Å was obtained with one such crystal grown in 5 m*M* uronate. However, no electron density was observed in the resulting maps that could be attributed to the added ligand. Soaking experiments were performed with crystals of AlgE grown in 7.8 MAG at 20°C as above. Crystallization plates containing mature crystals were opened (Li *et al.*, 2012 ▶), 1 µl of 5 or 50 m*M* di-mannuronate in 0.9 *M* sodium acetate and 0.1 *M* sodium cacodylate pH 6.5 was layered over the mesophase and the plates were resealed. After an incubation period of 90–120 min at 20°C, crystals were harvested and used for diffraction data collection to a resolution of 2.8 Å. The corresponding electron-density map was devoid of bound ligand.

### 2.4. Molecular-dynamics simulations of AlgE with citrate   

Molecular-dynamics simulations were performed using the *GROMACS* biomolecular simulation package (http://www.gromacs.org). All simulations were based on the highest, 1.9 Å resolution, structure (AlgE-1.9). The T8 loop, unresolved in this structure, was either modelled as a random coil (T8-disordered) or as an ordered T8 helix (T8-ordered) from AlgE-2.4A using *Modeller* (Sali & Blundell, 1993 ▶). These starting points were used to generate coarse-grained (CG) protein parameters. CG molecular-dynamics (CGMD) simulations used the Martini v.2.1 forcefield (Monticelli *et al.*, 2008 ▶) with an elastic network, in which harmonic restraints (force constant 100 kJ mol^−1^ nm^−2^) were applied to all C^α^ particles within 7 Å of each other. Self-assembly simulations were performed in which randomly orientated lipids spontaneously form a bilayer around the protein. The lipid mixture was chosen to be a simple model of a Gram-negative bacterial outer membrane, as discussed in §[Sec sec3]3. Cardiolipin parameters were taken from Dahlberg & Maliniak (2008 ▶). Charges were neutralized by adding ions, with an overall NaCl concentration of 0.15 *M*. The protein, lipids and solvent were pressure (101.3 kPa) and temperature (310 K) coupled to separate baths using the Berendsen algorithm (Berendsen *et al.*, 1984 ▶). A timestep of 20 fs was used. Analyses were performed using the *GROMACS* tools, *MDanalysis* (Michaud-Agrawal *et al.*, 2011 ▶) and locally written code.

The final frame from a 1 µs CGMD simulation was converted to an atomistic representation using a fragment-based approach as described elsewhere (Stansfeld & Sansom, 2011 ▶) with the atomistic protein structure aligned. A 1 ns simulation with the protein positionally restrained allowed the lipids to relax prior to production simulation. Atomistic simulations were performed using the GROMOS 53a6 force field following the same protocol as described elsewhere (Stansfeld & Sansom, 2011 ▶).

Citrate and alginate parameters were taken from the ATB database (Malde *et al.*, 2011 ▶). *VMD* (Humphrey *et al.*, 1996 ▶) and *PyMOL* were used for visualization.

### 2.5. CGMD simulations of AlgE and AlgK   

AlgK chain *B* (PDB entry 3e4b; Keiski *et al.*, 2010 ▶) was used as representative of the four chains. Initial attempts at docking of AlgE and AlgK using *ClusPro* were hampered by the lack of a membrane, leading to conformations that would not be possible *in vivo*. Therefore, to investigate potential inter­actions of AlgK with AlgE (with the T8 loop disordered), CGMD simulations were performed. Five independent starting configurations were generated in the following manner. A new set of AlgE protein CG parameters was generated using the final frame of the atomistic citrate–T8-disordered simulation. This was necessary as in CGMD simulations the secondary structure is pre-defined. Therefore, parameters with the T8 loop in a disordered conformation were generated. A CGMD bilayer self-assembly was run giving AlgE citrate–T8-disordered in the membrane. Next, the orientation of AlgK relative to the membrane was predicted using *Memembed* (Nugent & Jones, 2013 ▶). This led to an orientation in which AlgK is side-on to the membrane, only interacting with residues 128–130 buried in the hydrophobic region of the membrane. CG parameters for AlgK were generated as described for AlgE above using chain *B* of the crystal structure as the input. The relative orientations of AlgE and AlgK were generated by aligning the position of the membrane from CGMD and *Memembed*. Finally, the five starting points were generated by translating (40 × 40 Å in the bilayer plane and 10 Å away from the bilayer) and rotating (randomly about the bilayer normal) AlgK from the original position. In all five of the 1 µs simulations the two proteins were observed to interact.

It should be noted that the missing residues in the crystal structure were not modelled in for these simulations. CG simulations require pre-defined secondary and tertiary structures. These are unknown for the missing N-terminal residues and were excluded for the purpose of the AlgE–AlgK interaction simulations. It cannot be ruled out that the missing residues could play a role in the interaction between AlgE and AlgK. The lipidation site in AlgK is not included in the crystal structure either. The distance of the terminal N-residue (Gln12) is <20 Å from the lipid head-group region. This is within the distance that could be spanned by the 11 missing N-­terminal residues as a random coil. Future simulations could involve modelling the presence of the lipid anchor either by modelling in the missing residues and the lipidation site or by restraining the distance between the N-terminus and the lipid head groups.

### 2.6. Docking and MD simulations of alginate and AlgE   

Docking calculations were carried out using *AutoDock Vina* (Trott & Olson, 2010 ▶) with a search range (*x*, *y*, *z*) of 20, 20 and 50 Å. An octameric MGMGMGMG alginate molecule was used as the input for docking studies. The structure used was taken from the ATB website and was free to rotate in the calculations. Docking calculations were performed for the docking of octameric alginate to the AlgE-1.9 T8-ordered structure, the AlgE-2.4 *A* and *B* chains, and to simulation snapshots. The alginate was only observed to cross the pore in the fully open simulation snapshot. A representative low-energy docking pose of the alginate octamer within AlgE was chosen as the basis for a series of MD simulations. An unbiased MD simulation was performed for 100 ns, during which the alginate was stable in the pore. Steered molecular-dynamics (SMD) simulations were performed in which an imaginary harmonic spring is attached to a target group and a force applied to the spring relative to a reference point. SMD simulations used the *GROMACS* pull code with a pull rate of 0.5 m s^−1^. All other variables were identical to the previous MD simulations described above. A harmonic spring was attached to the centre of mass of the first M or G ring in alginate (depending on the direction of pushing, as shown in Supplementary Fig. S9^1^). In the simulations where the direction is reversed the spring is instead attached to the sugar unit at the opposite end of the alginate octamer. A force was then applied to the spring along the *z* axis away from a fixed reference group. The reference group was chosen to be the phosphate particles of the periplasmic leaflet of the lipid bilayer. A SMD simulation was also performed from the end point of the unbiased simulation described above. SMD simulation with protein positionally restrained used a force constant of 1000 kJ mol^−1^ nm^−2^ on all non-H atoms.

## 3. Results and discussion   

### 3.1. Protein production and characterization   

The protocol introduced in §[Sec sec2]2 generates naturally folded, membrane-integral AlgE (Rehm *et al.*, 1994 ▶). The protein was solubilized using β-OG and detergent-exchanged into LDAO before purification by affinity and size-exclusion chromatography for use in crystallization trials. Purified AlgE eluted on a size-exclusion column with the Gaussian profile of a monodisperse protein having an apparent molecular weight of 150 kDa (Supplementary Fig. S1Ai). Given a detergent micelle size of 17–22 kDa (Herrmann, 1962 ▶; Strop & Brunger, 2005 ▶) and a monomeric AlgE molecular weight of 51 kDa, the observed elution volume suggests that the protein may exist as a dimer or a trimer in solution. Another possibility is that the protein is a monomer, consistent with the crystal structure (see below) and *in vivo* cross-linking studies (Rehm *et al.*, 1994 ▶), but that the protein–detergent micelle includes cellular lipids that increase its apparent molecular weight. A loading-series analysis by SDS–PAGE showed that the protein has an estimated purity of ≥90% (Supplementary Fig. S1Aii). This was considered of good enough quality to proceed with crystallization trials and no further purification was attempted. AlgE displayed heat-modifiability (Tamm *et al.*, 2004 ▶), which is apparent as a shift on SDS–PAGE to an unfolded state with a higher apparent molecular weight. In the case of AlgE, a shift from 34 kDa (folded) to 51 kDa (unfolded) was observed (Supplementary Fig. S1Aii).

The electronic absorption, fluorescence and circular-dichroic properties of pure detergent-solubilized AlgE were as expected for a β-barrel protein with 15 tryptophans, 15 tyrosines and 21 phenylalanines (Supplementary Fig. S1B).

### 3.2. *In meso* crystallization and X-ray diffraction   

Initial trials performed using 9.9 MAG (monoolein) as the hosting mesophase lipid generated crystals that diffracted to 10 Å resolution. Subsequent rounds of optimization included screening the host lipid, buffer and pH, precipitant composition, salt and additive identity, protein concentration and temperature (Li *et al.*, 2011 ▶; Li, Lyons *et al.*, 2013 ▶; Li, Shah *et al.*, 2013 ▶). The best crystals were obtained with 7.8 MAG (Coleman *et al.*, 2004 ▶; Caffrey *et al.*, 2009 ▶) at 20°C (Supplementary Fig. S1C). These yielded three crystal forms in space groups *C*2, *P*2_1_2_1_2_1_, *P*2_1_ that diffracted to 1.9, 2.4 and 2.8 Å resolution, respectively. The corresponding structures, solved by molecular replacement with the published structure (Whitney *et al.*, 2011 ▶; PDB entry 3rbh) as the search model, are referred to herein as AlgE-1.9 (PDB entry 4afk), AlgE-2.4 (PDB entry 4b61) and AlgE-2.8 (PDB entry 4azl).

### 3.3. Overall structure   

**Figure 1:**
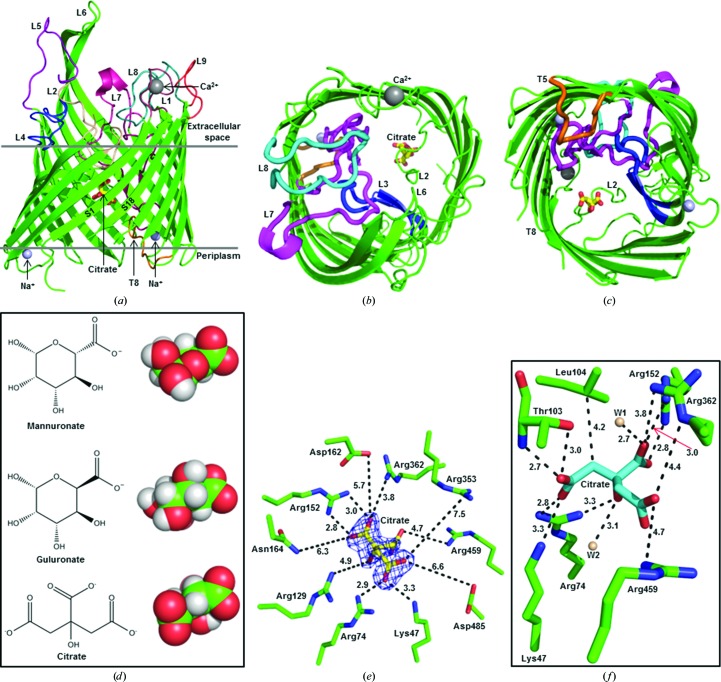
Crystal structure of AlgE. (*a*) Structure (ribbon model) of AlgE-1.9 viewed from the membrane plane. (*b*) As in (*a*), viewed from the extracellular space. (*c*) As in (*a*), viewed from the periplasm. (*d*) Chemical and space-filling structures of the mannuronate and guluronate components of alginate and of citrate. (*e*) Residues that form the electrostatic pore in which citrate sits in the lumen of AlgE-1.9. All residues are conserved (Whitney *et al.*, 2011 ▶). (*f*) Residues and water molecules (W1 and W2) that are within 5 Å of citrate in the lumen of AlgE-1.9. The distances in (*e*) and (*f*) are in Å.

The *in surfo* model of AlgE consists of an 18-stranded β-­barrel (Whitney *et al.*, 2011 ▶). The *in meso* structures, regardless of crystal form and space group, are also β-barrels (Figs. 1 ▶ *b* and 1 ▶ *c*) and are similar to the *in surfo* model (Supplementary Fig. S2). The *in surfo* and *in meso* structures were solved using protein that, in the former instance, started out as insoluble inclusion bodies. In the latter case, it was obtained from the membrane fraction. Thus, despite having entirely different initial states of folding and dispersion, and being crystallized by different methods, the final structures were alike. Relatedly, Hiller *et al.* (2010 ▶) reported that the structures of several membrane proteins obtained from native membranes and refolded from inclusion bodies are remarkably similar. The largest C^α^ r.m.s.d. was 0.86 Å observed between AlgE-2.4 chain *A* and 3rbh chain *B* (over 405 residues). Briefly, the structures include 18 antiparallel strands (S1–S18) that cross the membrane to varying degrees, nine extracellular loops (L1–L9) and eight periplasmic turns (T1–T8) (Supplementary Fig. S3). The loops and turns range in size from two to 43 residues (Supplementary Table S1), with L1 coordinating a calcium ion (Fig. 1 ▶). Both the N- and C-termini are in the periplasm. AlgE-1.9 has one molecule in the asymmetric unit. In contrast, the asymmetric units of AlgE-2.4 and AlgE-2.8 contain two molecules (molecules *A* and *B*). For all *in meso* structures, type I packing was observed (Supplementary Fig. S4), as is the norm for crystals grown in a lipidic mesophase environment (Caffrey *et al.*, 2012 ▶).

The AlgE crystal structures differ in resolution and model completeness. Missing residues map to the N-terminus, L1, L2, L5, L6, L9 and/or T8 (Supplementary Table S1 and Fig. S3) and correspond to regions in the protein that are disordered in the crystal. Disorder can reflect flexibility and mobility that may identify functional parts of the protein involved in alginate recognition, binding and transport, and sites of interaction with other proteins. With the exception of the N-terminus and L2, many of the missing residues in one model are present in at least one of the other models. This information, along with ligand docking and simulations studies, is used to describe a mechanism of action for AlgE (see below).

### 3.4. Bound citrate   

In the AlgE-1.9 structure, electron density located toward the mid-membrane plane of the barrel and a little off-centre in the barrel cavity was well fitted by a citrate molecule (Fig. 1 ▶). Citrate was present in the precipitant solution used for crystallization and is the likely source of the anion. It was not seen in the two other structures described in this study, in which T8 and L2 extend into the barrel with anionic side chains substituting, to some degree, for the bound citrate (Supplementary Fig. S5).

The cavity within the AlgE barrel has been proposed to act as a pore for alginate (Whitney *et al.*, 2011 ▶), a random, linear copolymer of 1,4-linked β-d-mannuronic acid (M) and its C-5 epimer α-l-guluronic acid (G). Interestingly, citrate is chemically and structurally similar to M and G (Fig. 1 ▶ *d*). Both contain at least one hydroxyl and one carboxyl group and both have similar molecular compositions (citrate, C_6_H_8_O_7_; M and G, C_6_H_10_O_7_) and molecular volumes (136 and 140 Å^3^, respectively, calculated using *Chem*3D *Ultra* 10.0; Mills, 2006 ▶). It is perhaps not surprising then that citrate is occasionally found as a ligand in the sugar and sugar polymer binding sites of proteins (Harrison *et al.*, 1994 ▶; Borrok *et al.*, 2007 ▶; Thamotharan *et al.*, 2011 ▶; Hansman *et al.*, 2012 ▶; Meekins *et al.*, 2013 ▶). We propose therefore that citrate acts as a mimic of the M and G components of alginate in AlgE-1.9.

The citrate molecule in AlgE-1.9 coordinates to a number of polar, apolar and cationic residues (Fig. 1 ▶ *f*). These include Lys47, Arg74, Thr103, Leu104, Arg152 and Arg362. Two structured waters are also involved. This type of interaction has been seen before in proteins that bind citrate (Russell *et al.*, 1997 ▶; Yue *et al.*, 2003 ▶; Sun *et al.*, 2010 ▶; Hansman *et al.*, 2012 ▶). The aforementioned cationic residues, along with Arg129, Asp162, Asn164, Arg353, Arg459 and Arg485, have been proposed to create a ring-like portal in AlgE that defines selectivity for the polyanionic alginate (Whitney *et al.*, 2011 ▶). When this ten-residue ring is highlighted in the AlgE-1.9 model, citrate sits neatly at its centre (Fig. 1 ▶ *e*). This suggests that citrate mimics the uronate components of alginate. Further, its location within the 10 Å diameter electropositive pore serves to identify it as a limiting structure through which alginate passes in its transit across the protein and the outer membrane.

We note that crystal packing can affect ligand binding (Cousido-Siah *et al.*, 2012 ▶). The magnitude of this effect in the current work is not easily gauged. Clearly, loop flexibility is influenced by packing, as shown by the *B*-factor distribution, and flexibility or a lack thereof can affect binding. *In vivo*, protein packing constraints of this type do not exist. However, the outer membrane is rich in lipopolysaccharide (LPS). Its large, bulky extracellular polysaccharide head group is likely to create a crowded and confining space at the membrane surface, which is where the extracellular loops of AlgE are found. In this regard, then, the crystal and *in vivo* environs of the protein mimic one another to some degree.

### 3.5. Disordered loops and a mechanism for alginate secretion   

**Figure 2:**
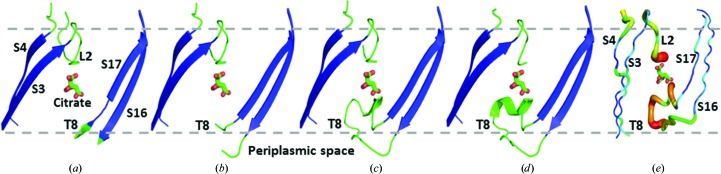
Extracellular gate (E-gate) and periplasmic gate (P-gate) and *B*-factor distribution in AlgE. (*a*–*d*) View from the membrane into the barrel of AlgE in different crystal forms highlighting different states of the E-gate (L2) and P-gate (T8) proposed to exist during alginate transport. (*a*) AlgE-1.9. (*b*) AlgE-2.4 chain *A*. (*c*) AlgE-2.4 chain *B*. (*d*) PDB entry 3rbh chain *B*. For reference, the citrate molecule observed in the electropositive pore in AlgE-1.9 (*a*) has been superimposed on the models in (*b*) and (*c*) as a point of reference. The relevant strands, loops and turns are labeled as described in Supplementary Fig. S3(*a*). The model in (*a*) best exemplifies AlgE with the P-gate (T8) open and the E-gate (L2) closed. The model in (*b*) illustrates AlgE with both the P-gate and E-gate closed. The models in (*c*) and (*d*) have the P-gate closed with the E-gate progressively more open. (*e*) Loop 2 (L2) and turn 8 (T8) have higher *B* factors than the rest of the protein. The structure is shown in putty representation and rainbow-coloured by *B* factor with hotter colours corresponding to higher *B*-factor values. For clarity, only L2 and T8 along with adjacent strands S3, S4, S16 and S17 are shown. The protein component in the figure is based on the AlgE-2.8 chain *B* model.

The interior of the barrel in all four AlgE structures accommodates L3 (155–161), L7 (334–376), L8 (404–424) and T5 (262–271) (Figs. 1 ▶ *b* and 1 ▶ *c*). Together, they fill and block approximately half the cavity from one lateral side of the barrel. The rest of the cavity would present an open, ∼5 Å diameter pore (from pore profile calculations using *HOLE*; Smart *et al.*, 1996 ▶) through the protein and across the membrane, were it not for L2 and T8. When L2 and T8 are in place they provide a tight seal, with a minimum constriction of 0.95 Å between the extracellular and periplasmic barrel ends, respectively. Hereafter, these loops are referred to as the extracellular gate (E-gate) and periplasmic gate (P-gate). Interestingly, we find that L2 and T8, while ordered in some crystal forms, are disordered in others (Fig. 2 ▶, Supplementary Fig. S3*b* and Table S1). Disorder, where electron density is either not observed or is ill-defined in the map, suggests that the corresponding loops, or parts thereof, are flexible and, in certain cases, may have relocated to reside outside the barrel. An example of a disordered loop is seen in AlgE-1.9, where electron density is missing for the entire T8 loop (Fig. 2 ▶ *a*, Supplementary Fig. S3). In the case of AlgE-2.4, which has two molecules in the asymmetric unit, T8 is disordered in molecule *A* (AlgE-2.4*A*) and is ordered in molecule *B* (AlgE-2.4*B*) (Supplementary Fig. S3 and Table S1). These two crystal forms are interpreted as examples of the alginate pore in which its periplasmic gate (P-­gate) is open and closed, respectively. Referring to the extracellular gate (E-gate) L2, while eight out of 28 residues in this loop are disordered in AlgE-1.9, the bulk of the loop is modelled and clearly blocks the pore (Figs. 1 ▶ *b*, 1 ▶ *c* and 2 ▶, Supplementary Table S1). This therefore represents the closed state of the E-gate. In the remaining crystal forms (AlgE-2.4*A*, AlgE-2.4*B*, AlgE-2.8*A*, AlgE-2.8*B* and PDB entry 3rbh) between ten and 17 residues in the E-gate are disordered (Fig. 2 ▶, Supplementary Fig. S3*b* and Table S1). We interpret these as representing states of the protein with less of the gate in place and, whilst still partially closed, they tend increasingly toward the open form of the E-gate.

**Figure 3:**
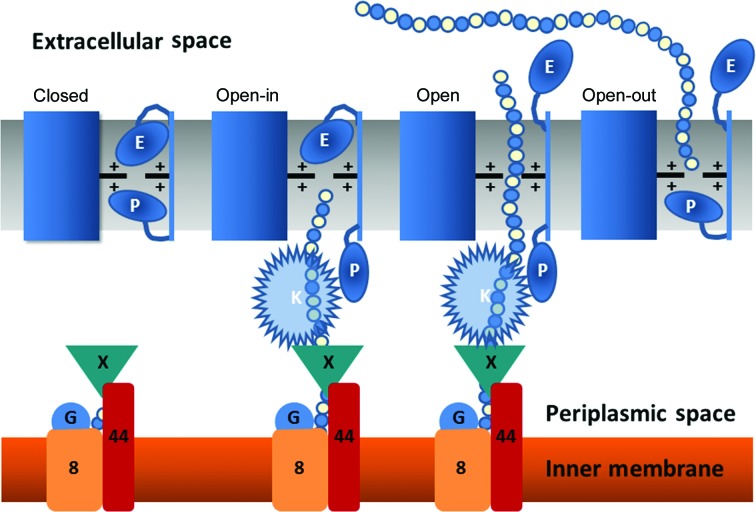
Cartoon representation of the proposed conformational states accessed by AlgE as alginate is moved across the outer membrane by the double-gate model. The mechanism includes four major states: closed, open-in, open and open-out. These correspond to conformations where the P-loop (loop T8) and E-loop (loop L2) gates are in the open or closed positions with respect to the cationic selectivity pore demarked with plus signs. The exopolysaccharide alginate is indicated as a string of blue and white circles emanating from the alginate-synthesizing machinery at the inner membrane. AlgK is proposed to associate with alginate and with the P-gate of AlgE to direct the polymer into the open-in AlgE pore. The alginate-synthesizing and secretion complex of Alg8, Alg44, AlgG and AlgX is arranged based on a published model (Rehman *et al.*, 2013 ▶). The peptidoglycan layer in the periplasmic space and AlgF, AlgI and AlgJ involved in alginate acetylation have been omitted for clarity. This cartoon is not drawn to scale.

It appears therefore that in this collection of six models we have an ensemble of structures that map a conformational landscape for alginate transport (Fig. 3 ▶). We refer to this as the double-gate model. To begin with, the ‘open-in’ form (P-gate open, E-gate closed) is primed to receive the nascent alginate polymer from the polymerizing machinery at its periplasmic side. At the same time, the pore is closed to bidirectional leakage across the membrane by the L2 or E-gate. This form is best represented in the collection by AlgE-1.9 (Fig. 2 ▶ *a*), which has citrate, the assumed alginate mimic, already halfway across the barrel sitting in the electropositive pore. Next on the conformational landscape is the ‘open’ form (P-gate open, E-­gate open), most closely represented by AlgE-2.4*A* (Fig. 2 ▶ *b*). *In vivo*, the open state would presumably have an alginate polymer threaded from the periplasm through the limiting electropositive pore extending out into the extracellular space. Thus positioned, alginate would block the pore and prevent leakage. As soon as the alginate polymerization has terminated and the final uronate approaches or has passed through the electropositive pore in the barrel, the third, ‘open-out’ conformation (P-gate closed, E-gate open) would emerge. This is most closely represented in the collection of structures by AlgE-2.4*B*, AlgE-2.8*A*, AlgE-2.8*B* and PDB entry 3rbh (Fig. 2 ▶, Supplementary Table S1). It is possible that alginate, as an exopolysaccharide, will eventually exit the barrel completely and move into the extracellular space, remaining loosely associated with the cell (Franklin *et al.*, 2011 ▶). In so doing, we speculate that the P-gate and E-gate re-enter the pore and become completely ordered. If such a fully ‘closed’ state exists, it has not been captured in the crystal forms reported to date. However, we note that closing either the P-gate or the E-gate should be sufficient to block the pore and prevent leakage.

### 3.6. In support of the proposed double-gate mechanism   

#### 3.6.1. Experimental   

Iodide-efflux measurements performed with pure AlgE reconstituted into liposomes suggest that the open conformation of the protein can form spontaneously (Whitney *et al.*, 2011 ▶). Deleting T8 entirely increased the iodide flux significantly and was interpreted as indicating that T8 (the P-gate) regulates anion passage across the membrane.

The *B* factors, which reflect thermal motion and static disorder, amongst other things, in the corresponding atom or groups of atoms, for residues in T8 and L2, when present, are higher than the average value for the entire protein (Fig. 2 ▶ *e*, Supplementary Fig. S6).

#### 3.6.2. Molecular-dynamics simulations   

Molecular-dynamics (MD) simulation studies have been performed that support and extend elements of the proposed mechanism for alginate transport. These began with an assessment of the stability of the highest resolution structure AlgE-1.9 in a model of a bacterial outer membrane. An initial configuration was generated by coarse-grained (CG) self-assembly simulation, allowing a tertiary lipid mixture of phosphatidylethanolamine, phosphatidylglycerol and cardiolipin (CL; 4:2:1 molar ratio) to spontaneously form a bilayer within which the protein inserted. This is a simplified model of the asymmetric outer membrane of Gram-negative bacteria in which the inner leaflet is composed of phospholipids (including CL) while the outer leaflet is almost exclusively composed of LPS (Osborn, 1969 ▶). CL and LPS are anionic, with total negative charges of −2 and −4, respectively. We therefore expect CL to bind in similar sites to LPS. This approach has been used successfully before (Baaden & Sansom, 2004 ▶). The protein remained stable in the bilayer for the duration of each simulation. Analysis of an extended 1 µs CGMD simulation indicated some local bilayer deformation close to the protein, with no significant increase in deformation in the region of the shorter S5 and S6 β-strands (Supplementary Fig. S7). This is consistent with the suggestion that Phe187 and Tyr190 on L4 extend the hydrophobic surface of the protein, allowing it to function in the membrane in a monomeric form rather than in the trimeric state observed for OmpF (Cowan *et al.*, 1992 ▶), for example, that has equivalent short β-­strands. Further analysis of protein–lipid interactions yielded specific CL ­protein binding sites (Supplementary Fig. S7). These CGMD simulations provided an equilibrated protein-bilayer system for conversion to atomistic resolution (preserving the crystal structure of the protein) for further analysis (Stansfeld & Sansom, 2011 ▶; Supplementary Fig. S7).

#### 3.6.3. Molecular dynamics involving citrate   

The simulations presented here are based on the highest resolution structure AlgE-1.9, with the T8 loop (P-­gate) inserted either as random coil (referred to here as T8-disordered) or as an ordered structure (T8-ordered; see §[Sec sec2]2 for full details). Three atomistic simulation systems were generated to assess the behaviour of the T8 loop and how citrate interacts with the protein. A simulation of the citrate-bound form, with the T8 loop initially in the ordered conformation (citrate–T8-ordered), was performed to evaluate the influence of the bilayer on the proposed P-gate ‘closed’ conformation. An equivalent simulation with T8 modelled initially as a random coil, corresponding to the P-gate ‘open’ state (citrate–T8-disordered), was performed for comparison. Finally, a T8-­ordered apo simulation was performed in the absence of citrate (apo T8-ordered).

**Figure 4:**
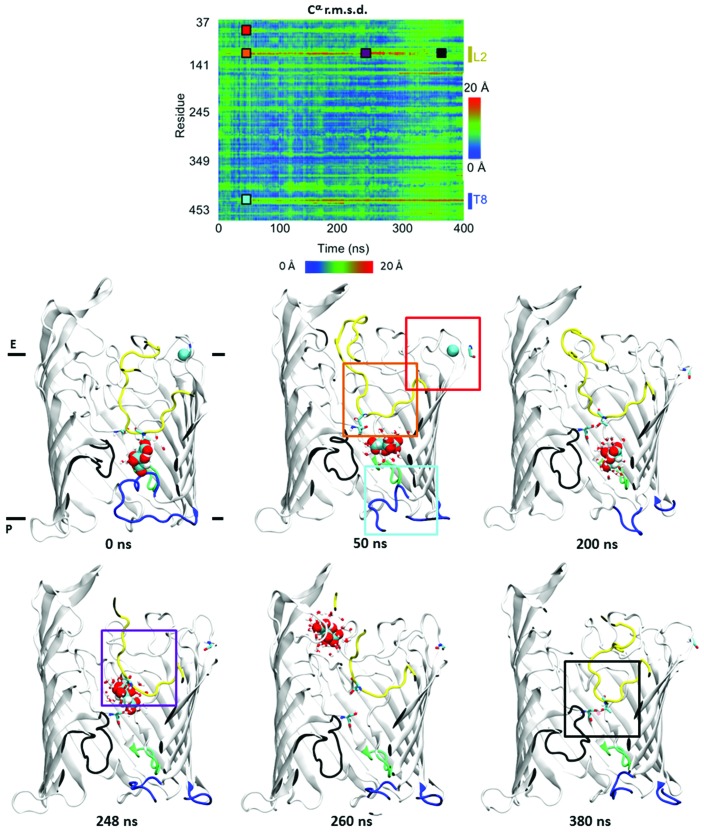
Motion of protein loops during the exit of the citrate molecule from the binding site through the extracellular gate. The 1.9 Å resolution citrate-bound crystal structure with the T8 loop modelled in an ordered conformation was used in this simulation. Upper panel: time evolution of the root-mean-squared deviation of protein backbone atoms. The T8 and L2 loop regions are indicated. Coloured squares identify key motions of the protein. Lower panels: snapshots of citrate motion through the E-gate. The approximate location of the extracellular (E) and periplasmic (P) sides of the membrane are indicated. Coloured boxes correspond to the squares in the upper panel. The protein is shown in cartoon representation with the loops coloured as follows: T8, blue; L2, yellow; L3, black; L6, green. The citrate molecule is shown in space-filling representation. Citrate passes through the E-gate at ∼250 ns. Following citrate exit, the L2 loop moves back towards its initial conformation (black square), closing the pore once again.

Distinct behaviour was observed in the three simulations, each providing further insight into the proposed mechanism of alginate transport. We first present the results of the citrate–T8-ordered simulation, in which the citrate moves from the periplasmic binding site through the E-gate before exiting the protein completely. The mechanism of citrate ‘export’ may be broken down into several key steps, which are highlighted in Fig. 4 ▶ and shown in Supplementary Movie 1.

Three main conformational changes occurred during the first 50 ns of the simulation. Firstly, the T8 loop began to move away from the citrate molecule towards the periplasm. Concomitantly, there was an increase in motion of the citrate molecule, followed by a movement of the L2 loop and a loss of Ca^2+^ from L1. Subsequently, smaller diffusive motions occurred until the citrate approached and then passed through the E-gate. In the process, a hydrogen-bonding interaction between the conserved residues Thr103 (L2) and Ser351 (L7) was broken. When the E-gate is closed these residues are hydrogen-bonded together, closing the pore. As the citrate approached Thr103 it formed hydrogen-bonding interactions, breaking the initial hydrogen-bond ‘lock’ with Ser351. At this point, the citrate exited the pore and, in so doing, interacted with Arg74, Lys47, Thr103, Arg129, Arg152 and Arg353 that line the pore. After citrate left the pore, the L2 loop recovered its original conformation in the pore, closing the E-gate. In contrast to L2, the T8 loop moved towards its original location but did not completely recover its initial conformation on the timescale of the simulation. The distance separating the locking residues Thr103 and Ser351 may be used as a measure of gate opening. Supplementary Fig. S8 shows how water flux varied with opening and closing of the E-gate. These simulations show that the AlgE pore can open from both extracellular and periplasmic ends to a degree sufficient for citrate (and thus alginate) to pass through.

Distinctly different behaviour was observed in the citrate–T8-disordered simulation. The T8 loop acquired some β character and became more compact within the first 50 ns, but did not adopt any of the ordered α-helical structure from the initial random-coil configuration. This is not surprising, as protein disordered-to-folded events are not generally expected to occur on a submillisecond timescale. The citrate remained in the binding site for 50 ns before partially and then fully exiting the initial binding site, this time towards the periplasmic side after 90 ns, only interacting with a single residue, Arg154, along the way. Together, these two simulations involving citrate demonstrate that citrate is able to move in either direction from its initial binding site. This has implications for the model of alginate transport. It is to be expected, however, that the interactions of a polymer with a highly charged pore are more complex than those experienced by a simple citrate molecule and that such interactions may influence the free-energy landscape of alginate transport, and hence the ‘directionality’ of the latter in terms of the likely energetic barriers encountered.

Intriguingly, in the apo T8-ordered simulation the T8 loop remained in the ordered conformation (P-gate closed) for the duration of a 100 ns ATMD simulation. This result suggests that interaction with the alginate subunit mimic citrate, perhaps modulated by another component of the pathway, increases the rate of opening of the P-gate. The closed P-gate equates to a lower rate of water passing through the pore, with ∼1.4 water molecules per nanosecond traversing the pore compared with 3.5 molecules per nanosecond for the citrate–T8-disordered simulation. This is consistent with experimental T8 deletion data in which removal of the T8 loop led to increased ion flux (Whitney *et al.*, 2011 ▶).

#### 3.6.4. Molecular dynamics and docking involving AlgK   

**Figure 5:**
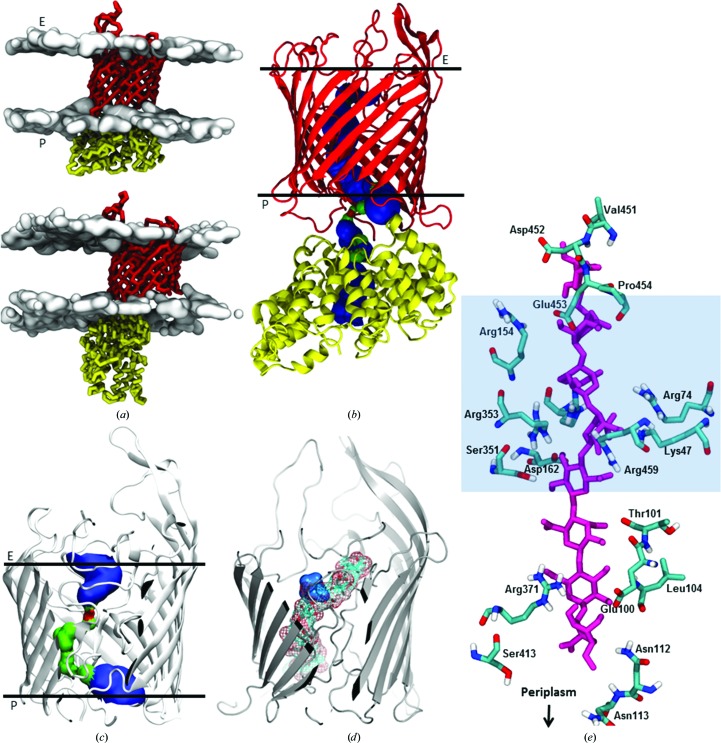
(*a*, *b*) Interactions of AlgE with AlgK (left-hand panels) and alginate (right-hand panels). (*a*) The two observed AlgE–AlgK interactions in the presence of the membrane as generated by CGMD simulations. The end-to-end pose (top) was observed in two of five simulations, with an end-to-side pose (bottom) observed in the remaining three. (*b*) Pore profile of the end-to-end configuration in (*a*) converted to atomistic resolution. Blue corresponds to an open channel (radius > 2.3 Å), green corresponds to single-file water (1.2 Å ≤ radius ≤ 2.3 Å) and red corresponds to a fully closed channel (radius < 1.2 Å). The only constriction observed is in the mobile loop region. (*c*) Typical pore profile of the static AlgE crystal structures, highlighting the constriction around the Thr103–Ser351 region that prevents the passage of alginate. Surface coloured as in (*b*). (*d*) Alginate docked into a protein structure taken from a frame during simulation of citrate exit, where the constriction site is fully open. The position of citrate within this region is shown as a blue surface. Alginate is shown as a mesh with O atoms coloured red and C atoms coloured cyan. (*e*) Key protein–alginate interactions observed during an unbiased simulation of alginate within the pore beginning from the docked pose shown in (*c*). Alginate remains stable within the pore for the duration of the simulation. The cationic citrate-binding site region is shaded.

It has been proposed that T8 in the P-gate open state may interact with AlgK, a periplasmic lipoprotein known to be essential for alginate secretion (Keiski *et al.*, 2010 ▶; Whitney *et al.*, 2011 ▶; Rehman *et al.*, 2013 ▶). Fig. 5 ▶ shows the predicted relative orientations of AlgK (PDB entry 3e4b) and AlgE T8-disordered on and in the membrane. These are based on the predicted membrane alignment of AlgK (using *Memembed*; http://bioinfadmin.cs.ucl.ac.uk/) and the position generated *via* the CGMD simulation of AlgE. We used this initial configuration to generate five different starting points for exploratory CGMD simulations, with AlgK in different relative orientations with respect to AlgE in each case (see §[Sec sec2]2). In three of the five trials the AlgK interacted with AlgE in a ‘side-on’ (Fig. 5 ▶) orientation, similar to the initial orientation predicted from *Memembed*, with minimal interaction with the disordered T8 loop. In the remaining two simulations the AlgK rotated 90° to assume an ‘end-on’ position on AlgE. This binding mode created a pore that extends from AlgE into AlgK and through which alginate may still be docked (see below). To the best of our knowledge, this binding mode has not been described previously. This orientation effectively extends the length of the pore through which alginate must pass. We suggest therefore that AlgK plays a role in pre-ordering the alginate polymer before being threaded into AlgE. This hypothesis is supported by the fact that the pore in AlgK is larger than that through AlgE. Of course, the absence of N-terminal residues (including the lipidation site) in the original AlgK crystal structure, along with the variation in the conformation of the N-terminal helix means that further work will be required to fully characterize the mode of this proposed AlgE–AlgK interaction.

#### 3.6.5. Docking alginate   

To further investigate alginate transport, several docking calculations were run. An octameric form of alginate (MGMGMGMG) was chosen as it is of sufficient length (∼40 Å) to entirely cross the pore of AlgE. Docking calculations were carried out for all models (see §[Sec sec2]2). Uniformly, the octamer was found to dock in one of two positions, in the periplasmic or extracellular end of the barrel (Fig. 5 ▶ *d*), with similar energetics. The octamer did not thread through the pore in any of the docking runs, consistent with the constriction at the E-gate in each trial (minimum pore diameter <4 Å; Fig. 5 ▶). Therefore, as may be anticipated from the simulations with citrate, the E-gate is required to open fully to allow the octamer, and by extension alginate, to pass through.

To compare the proposed fully open conformation obtained during the simulation with citrate, a frame from the ‘citrate-export’ simulation was chosen in which the citrate is passing by the E-gate. As noted, the molecular size, shape and volumes of citrate and the M/G subunits of alginate are similar. We expected therefore that the selected conformation corresponded to one through which alginate should be able to pass. Docking studies confirmed this, with the majority of the docking poses corresponding to those in which the octamer passed through the pore (Fig. 5 ▶ *e*). It is not known whether uronates are added onto nascent polymer at the reducing or the nonreducing end. No preference was observed for the glycosidic 1–4 or 4–1 alginate orientation, with ∼50% of the top poses corresponding to each, and the top docking pose corresponding to −10.4 kcal mol^−1^ for each orientation.

#### 3.6.6. MD simulations with alginate in the AlgE pore   

In an unbiased simulation the alginate octamer in the pore equilibrated rapidly (<10 ns) to a stable configuration that was maintained for the remainder of the 100 ns simulation. The ten residues identified as forming the electropositive ring, along with others in the pore, interact with the octamer (Fig. 5 ▶ *f*). This demonstrates that alginate does not diffuse passively across the pore, at least on timescales accessible to unbiased atomistic MD simulations. Accordingly, energy is required for alginate export. Presumably, this is provided by the polymerizing machinery that extends the alginate molecule, one uronate monomer at a time, in the direction of the pore. To mimic this behaviour, steered MD (SMD) simulations were carried out, in which a directional biasing force was applied to either ‘push’ or ‘pull’ the alginate through the pore from the periplasmic side to the extracellular side (Supplementary Fig. S9). The pushing simulation corresponds to a situation in which the force moving along the alginate would come from the alginate-synthesizing machinery. The pulling simulation provides information on the final stages of alginate translocation, once the polymer synthesis has been terminated. This would correspond to a situation in which the entire alginate polymer exits the AlgE pore into the extracellular space. It should be noted that it is currently unknown how this final stage of alginate export occurs and whether the polymer remains threaded through the pore of AlgE or leaves the pore to interact with the extracellular loops and/or LPS. The magnitude of the applied force gives an indication of the energy required to move the alginate through the pore. Motion was not smooth in either the pushing or pulling cases, indicating that there are local energy minima for alginate within the pore. SMD simulations in the reverse direction (extracellular to periplasmic direction) led to similar force profiles. Thus, AlgE itself does not appear to impart any directionality to alginate transport. A further SMD simulation was begun following the unbiased simulation of alginate in the pore to provide a more equilibrated starting point. This export SMD simulation is shown in Supplementary Movie 2. It is apparent that the protein forms a largely well defined pore through which the alginate twists and turns, although the process may be aided by ‘breathing’ motions of the protein. To investigate the latter possibility, a SMD simulation in which the protein conformation was fixed (by applying positional restraints to all non-H atoms) was performed. This revealed that the alginate was not able to move, implying that small backbone motions are required for transport.

In this study, we have shown that the β-barrel structure of AlgE crystallized using native membrane-derived protein was the same as that refolded from inclusion bodies. The study included several crystal forms that differed with regard to the presence or absence of electron density for loops L2 and T8 at either end of the barrel. This variability has been interpreted as indicating flexibility in the corresponding loops, strengthening the proposal that these act as gates in the core of the barrel to regulate alginate export whilst minimizing leakage. Citrate was found in the selectivity filter of one crystal form. Given that citrate and the monomeric units that make up alginate are similarly constituted, its location in the barrel was interpreted as indicating a route taken by alginate through the pore in the process of being secreted. Computational studies supported and extended these experimental observations. They demonstrated that the L2 and T8 loops are flexible and may open sufficiently to allow a citrate/alginate molecule to pass during unbiased simulation. However, motion of the T8 loop that opens the P-gate appeared to require the presence of citrate and/or another component of the pathway. The periplasmic protein AlgK has previously been identified as a likely candidate (Whitney *et al.*, 2011 ▶), and was shown in coarse-grained simulation studies to complex with the periplasmic domain of AlgE. Recent pulldown and mutual stability measurements indicate that AlgK binds to AlgE, corroborating the modelling results (Rehman *et al.*, 2013 ▶). Future computational studies using ever more realistic bacterial membrane compositions (Piggot *et al.*, 2011 ▶) will provide further insight into the mechanism of alginate export. Based on the simulations discussed here, we may expect LPS to play a role in stabilizing the E-gate open configuration, with the extended form of the extracellular loop L2 interacting with LPS, as proposed previously (Whitney *et al.*, 2011 ▶). 

## Supplementary Material

PDB reference: AlgE, 4afk

PDB reference: 4b61

PDB reference: 4azl

Supporting Information.. DOI: 10.1107/S1399004714001850/be5250sup1.pdf

Supplementary Movie 1. DOI: 10.1107/S1399004714001850/be5250sup3.mpg

Still Image and Legend for Movie 1. DOI: 10.1107/S1399004714001850/be5250sup4.pdf

Supplementary Movie 2. DOI: 10.1107/S1399004714001850/be5250sup5.mov

Still Image and Legend for Movie 2. DOI: 10.1107/S1399004714001850/be5250sup6.pdf

## Figures and Tables

**Table 1:** Data-collection and refinement statistics Values in parentheses are for the highest resolution shell.

	AlgE-1.9 (PDB entry 4afk)	AlgE-2.4 (PDB entry 4b61)	AlgE-2.8 (PDB entry 4azl)
Data collection			
Space group	*C*2	*P*2_1_2_1_2_1_	*P*2_1_
Unit-cell parameters			
*a* (Å)	57.25	61.67	47.09
*b* (Å)	74.42	71.46	245.76
*c* (Å)	115.53	240.29	47.13
α = γ (°)	90	90	90
β (°)	101.56	90	104.36
Wavelength (Å)	0.97934	0.97934	0.97934
Resolution (Å)	113.18–1.90 (1.97–1.90)	55.64–2.40 (2.53–2.40)	44.90–2.80 (2.95–2.80)
*R*_merge_	0.14 (0.35)	0.09 (0.55)	0.22 (0.91)
*R*_r.i.m._	0.15 (0.45)	0.10 (0.58)	0.28 (1.15)
〈*I*/σ(*I*)〉	15.7 (2.9)	6.9 (1.8)	5.8 (2.0)
Completeness (%)	97.7 (84.9)	98.6 (99.9)	93.2 (95.3)
Multiplicity	6.4 (2.5)	9.3 (9.4)	2.7 (2.7)
Wilson *B* factor (Å^2^)	15.42	35.72	36.86
Refinement			
Resolution (Å)	19.77–1.90	48.87–2.40	44.89–2.80
No. of reflections	35580	45680	23738
*R*_work_/*R*_free_	0.163/0.209	0.221/0.244	0.237/0.280
No. of atoms			
Total	3999	7440	6941
Protein	3415	6903	6701
Ion	3	9	2
Ligand	339	324	189
Water	242	204	49
No. of residues	430	871	857
Chain (residues present)	*A*, 37–108, 117–438, 455–490	*A*, 39–105, 116–440, 454–490; *B*, 38–105, 117–490	*A*, 40–103, 121–290, 298–490; *B*, 39–104, 120–290, 298–490
*B* factors (Å^2^)			
Protein	21.4	42.0	44.4
Ion	38.9	60.5	66.0
Ligand/ion	44.4	50.5	44.5
Water	31.1	38.2	27.3
R.m.s. deviations			
Bond lengths (Å)	0.010	0.019	0.006
Bond angles (°)	1.344	1.109	1.218
Ramachandran plot			
Favoured region (%)	97.41	96.85	97.45
Allowed region (%)	2.35	2.92	2.31
Outlier region (%)	0.24	0.23	0.24
*MolProbity* clashscore	7.31	10.08	7.69

## Footnotes

1 Supporting information has been deposited in the IUCr electronic archive (Reference: BE5250).
