# Supplementary material for: A conformational landscape for alginate secretion across the outer membrane of Pseudomonas aeruginosa
Source: Acta Crystallogr D Biol Crystallogr. 2014 Jul 25;70(Pt 8):2054–68. doi: 10.1107/S1399004714001850 (PMC4118822; doi:10.1107/S1399004714001850)
Supplement: Supplementary file 1 [file d-70-02054-sup1.pdf]

Acta Crystallographica

Section D

Volume 70 (2014)

Supporting information for article:

A conformational landscape for alginate secretion across the outer membrane of *Pseudomonas aeruginosa*

Jingquan Tan, Sarah L. Rouse, Dianfan Li, Valerie E. Pye, Lutz Vogeley, Alette R. Brinth, John C. Whitney, P. Lynne Howell, Mark S. P. Sansom and Martin Caffrey

Supporting Information

A Conformational Landscape for Alginate Secretion Across the Outer Membrane of *Pseudomonas aeruginosa*

Jingquan Tan [1,a], Sarah L . Rouse [2,a], Dianfan Li [1,a], Valerie E. Pye [1,3], Lutz Vogeley [1], Alette R. Brinth [1], John C. Whitney [4], P. Lynne Howell [4], Mark S. P. Sansom [2], Martin Caffrey [1*]

[1]Schools of Medicine and Biochemistry & Immunology, Trinity College, Dublin, Ireland; [2]Department of Biochemistry, University of Oxford, South Parks Road, Oxford, UK; [3] Current Address: Clare Hall Laboratories, Cancer Research UK, Herts., UK; [4]Program in Molecular Structure & Function, The Hospital for Sick Children, Toronto, Canada & University of Toronto, Toronto, Ontario, Canada.

[a]These authors contributed equally to this work.

*Corresponding author. Email: martin.caffrey@tcd.ie

Supplementary Figures and Tables

Fig. S1-S9

Table S1

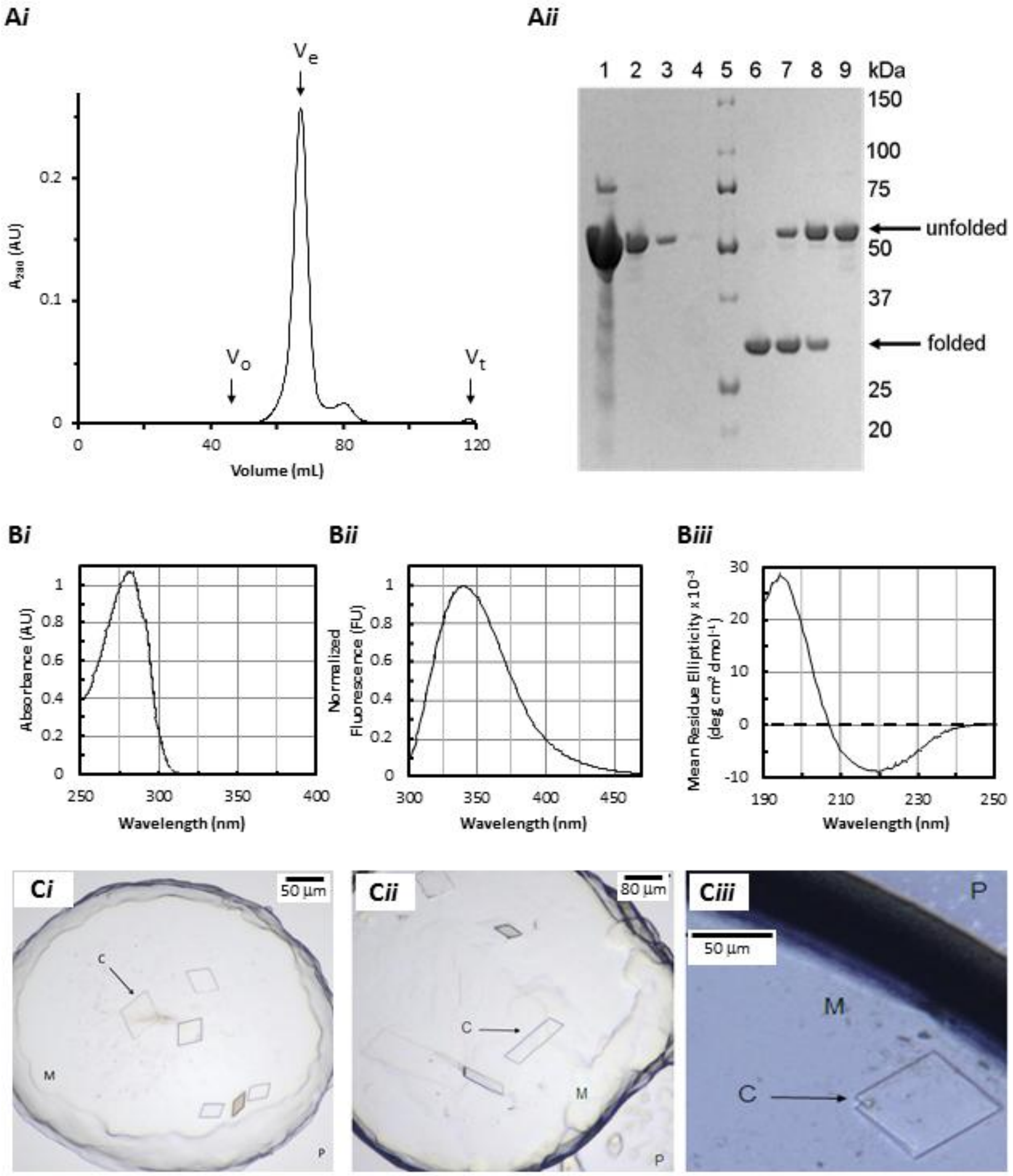

Figure S1 Purification, spectroscopic characterization and crystallization of AlgE. (*Ai*) Size exclusion chromatographic analysis. V_o, V_e and V_t mark the void, elution and total column volumes, respectively. The near Gaussian-shaped elution profile is consistent with a monodisperse protein preparation suitable for crystallization trials. (*Aii*) SDS-PAGE analysis. Protein purity of AlgE post-size exclusion chromatography was estimated at >90 % based on a loading series analysis with Coomassie−Blue staining. The following AlgE loadings were used: 100 µg, 10 µg, 1 µg and 0.1 µg

in lanes 1-4, respectively. Molecular weight standards are in lane 5. The hallmark heat modifiability of outer membrane β-barrel proteins is illustrated in lanes 6-9 where AlgE samples were incubated at 50 °C for 0 min, 5 min, 20 min and 120 min, respectively, before being electrophoresed. (*B*) Spectrophotometric analysis of AlgE. (*Bi*) UV-visible, (*Bii*) fluorescence and (*Biii*) circular dichroism spectra of AlgE in a detergent micellar solution at 20 °C. Spectra were recorded in Buffer B at 0.5, 0.1 and 1 mg AlgE/mL, respectively. An excitation wavelength of 280 nm was used for fluorescence data collection. Maximum absorbance and fluorescence emission was observed at 281 and 339 nm, respectively. The amino acid composition of AlgE is as follows: Ala, 37, 8.1%; Arg, 33, 7.2%; Asn, 20, 4.4%;, Asp, 46, 10.0%; Cys, 0, 0%; Gln, 20, 4.4%; Glu, 26, 5.6%; Gly, 51, 11.1%; His, 11, 2.4%; Ile, 16, 3.5%; Lys, 13, 2.5%; Leu, 36, 7.9%; Met, 4, 09%; Phe, 21, 4.6%; Pro, 14, 3.1%; Ser, 28, 6.1%; Thr, 34, 7.4%; Trp, 15, 3.3%; Tyr, 15, 3.3%; Val, 18, 3.9%. Analysis of the circular dichroism spectrum revealed a β-strand, α-helix, turn and coil content of 50, 20, 6 and 24 %, respectively, in reasonable agreement with the crystal structure. (*C*) Crystals of AlgE growing in the lipidic mesophase formed by 7.8 MAG at 20 °C. Trials were conducted as described (refs) using 10 mg AlgE/mL. The precipitant included (*Ci*) 0.1 M Bis-Tris, pH 7.0, 0.1 M sodium citrate, 21 %(v/v) PEG400; (*Cii*) 0.1 M sodium cacodylate, pH 6.5, 0.9 M sodium acetate; and (*Ciii*) 0.1 M Tris-HCl, pH 7.5, 0.1 M sodium citrate, 18 %(v/v) PEG 400. The crystal in (*Ciii*) gave best resolution to 1.9 Å. It continued to grow en route to the synchrotron and at time of data collection measured 150 μm X 150 μm. C, M and P refer to crystal, mesophase and precipitant, respectively. The image in (*Ciii*) was recorded with a different imager to that used in (*Ci*) and (*Cii*) which accounts for the difference in background color.

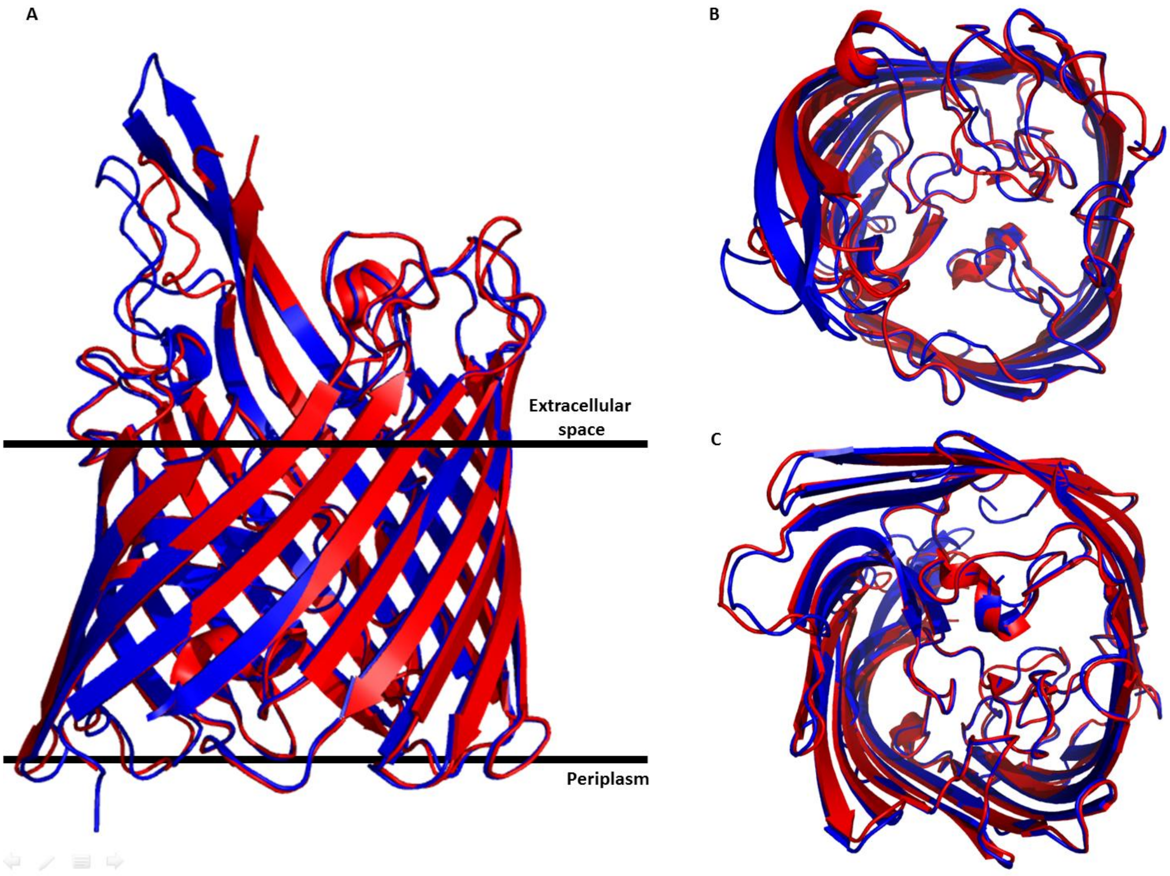

Figure S2 Superposition of the *in surfo* and *in meso* AlgE structures. Chain B of AlgE-3RBH (red) and AlgE-2.4B (blue) were used for comparison because they represent the most complete structures. Structures are shown viewed from within the membrane (*A*), from outside the cell (*B*) and from the periplasm (*C*).

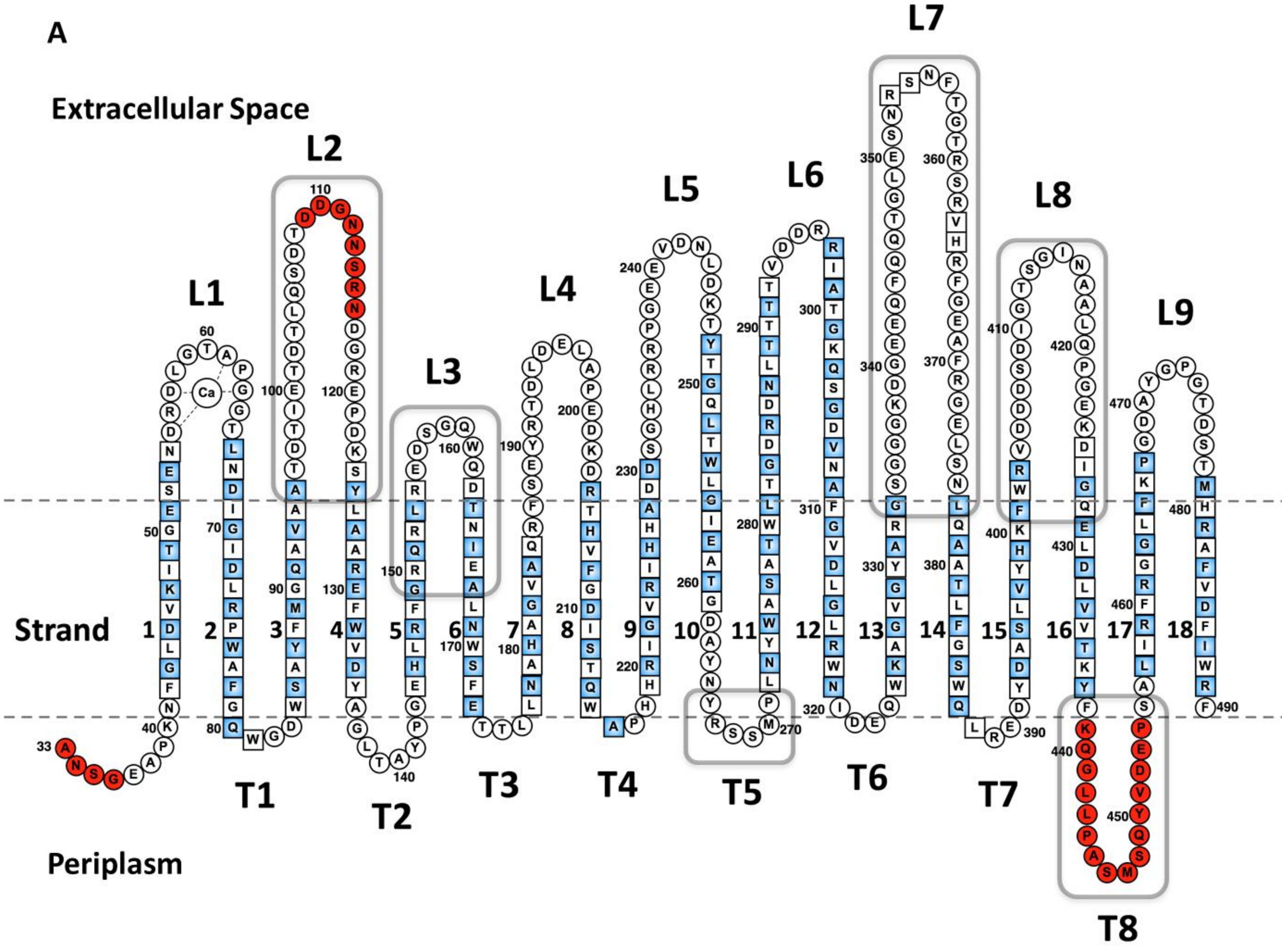
A
Extracellular Space
L1
L2
L3
L4
L5
L6
L7
L8
L9
Strand
1
2
3
4
5
6
7
8
9
10
11
12
13
14
15
16
17
18
T1
T2
T3
T4
T5
T6
T7
T8
Periplasm
Ca

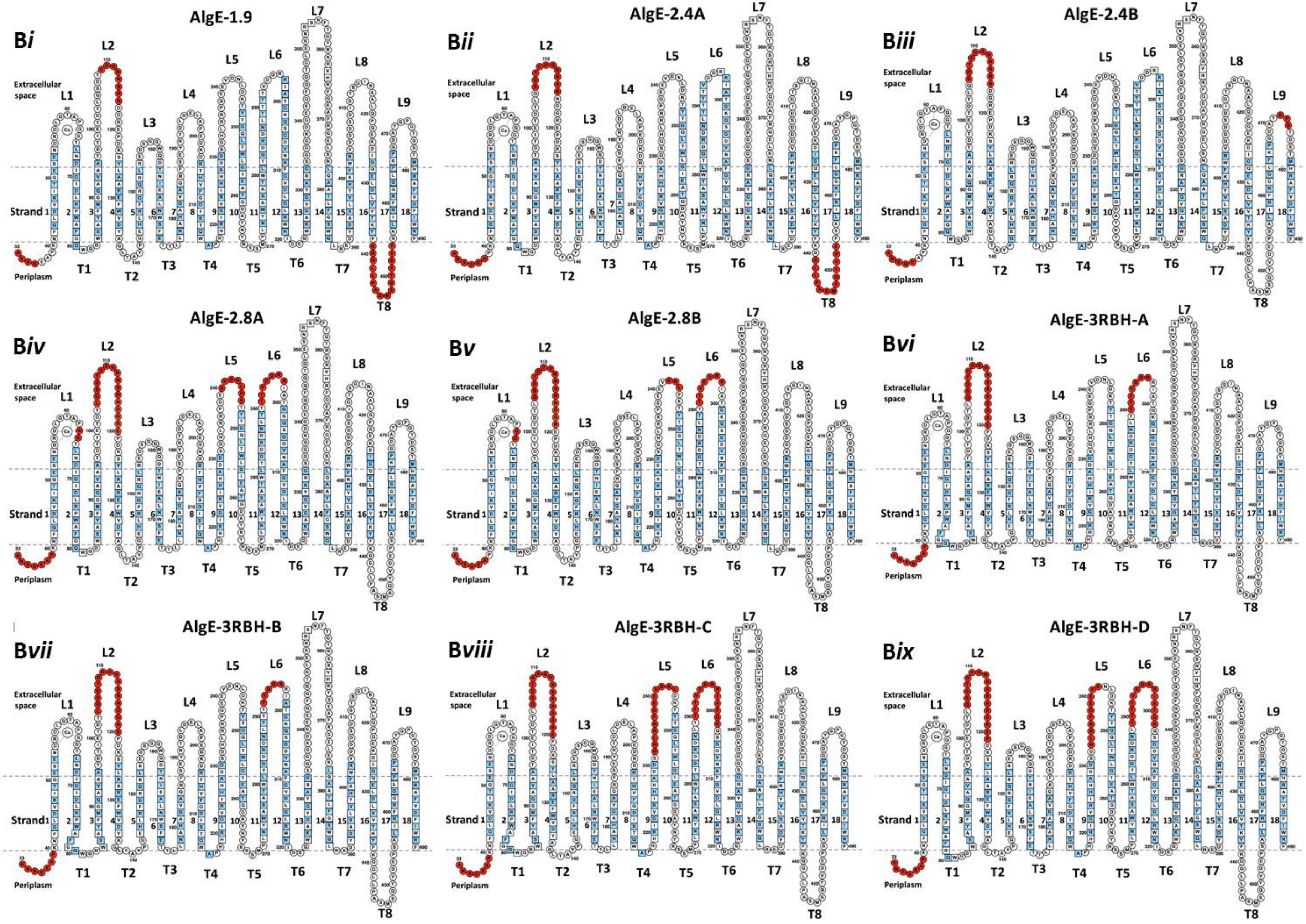
B*i*
AlgE-1.9
B*ii*
AlgE-2.4A
B*iii*
AlgE-2.4B
B*iv*
AlgE-2.8A
B*v*
AlgE-2.8B
B*vi*
AlgE-3RBH-A
B*vii*
AlgE-3RBH-B
B*viii*
AlgE-3RBH-C
B*ix*
AlgE-3RBH-D
Extracellular space
Periplasm
Strand
L1
L2
L3
L4
L5
L6
L7
L8
L9
T1
T2
T3
T4
T5
T6
T7
T8

Figure S3 Membrane topology, sequence and secondary structure of *in meso* and *in surfo* AlgE models. (*A*) The model shown is based on the AlgE-1.9 *in meso* crystal structure. Squares correspond to residues in strands that, for the most part, constitute barrel staves. Blue shading identifies residues whose side chains face the lumen of the barrel. Residues that lack electron density are identified by red shading. Turns and loops are denoted T and L, respectively. β-Strands are numbered sequentially toward the membrane mid-plane. The dashed horizontal lines show the approximate location of the membrane boundary as calculated in the PPM server (http://opm.phar.umich.edu/server.php) (Lomize *et al.*, 2012). The representation shown has been adjusted for clarity and legibility by orienting strands vertically and aligning them sequentially left to right. Note that the S5-L3-S6 segment between residues R150 and E166 folds into the lumen of the barrel and that sequential residues K129 and E130 both have side chains facing the lumen. Gray, round cornered boxes identify loops and turns that, when present in density, fold back into the barrel lumen. (*B*) Membrane topology, sequence and secondary structure comparison of *in meso* and *in surfo* AlgE models. Red coloring identifies residues that lack electron density and are assumed to be in disordered segments. Scanning across the panels it is apparent that the P-gate (T8) is present and presumed closed in models in *iv*-*ix*. The P-gate is open in *ii* and *iii*. By contrast, across the series from *ii* to *ix* the E-gate (L2) has varying stretches of the loop disordered corresponding to different degrees to which the gate is opened/closed. *i*-*v* correspond to *in meso* models reported in this work. *vi*-*ix* correspond to Molecules A-D in the *in surfo* model, 3RBH (Whitney *et al.*, 2011).

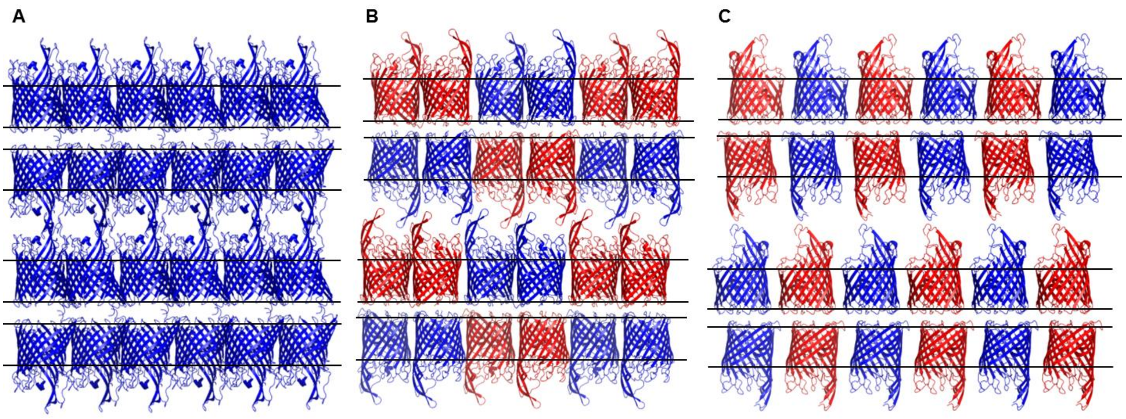

Figure S4 Packing arrangement in crystals of AlgE colored by asymmetric unit. (*A*) Crystal packing in AlgE-1.9, space group C2, which has one molecule per asymmetric unit. (*B*) Crystal packing in AlgE-2.4, space group $P2_12_12_1$, which has two molecules per asymmetric unit. (*C*) Crystal packing in AlgE-2.8, $P2_1$, which has two molecules per asymmetric unit. Packing is of Type I, as observed with other structures solved with crystals grown by the *in meso* method (Caffrey *et al.*, 2012).

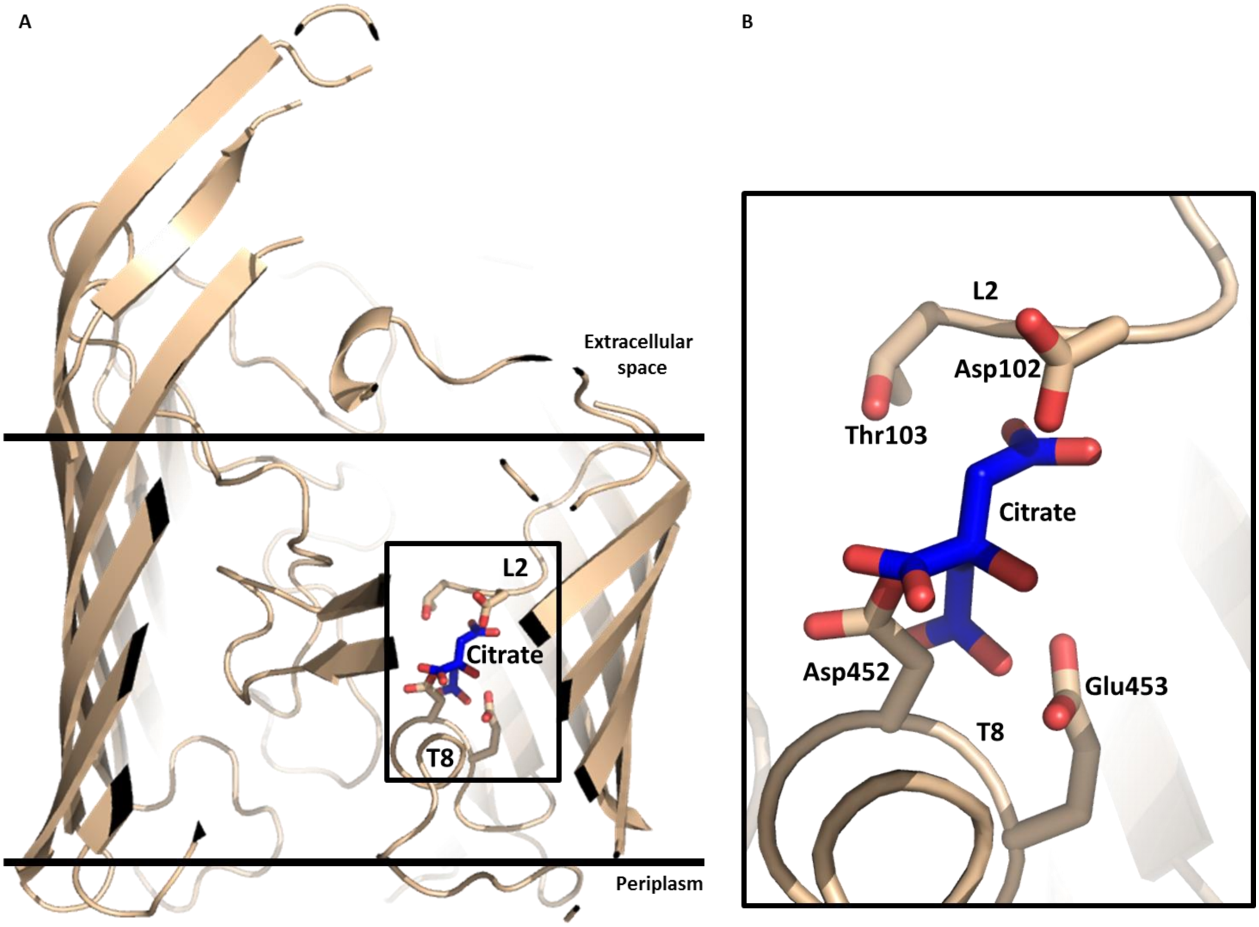

Figure S5 Superposition of the citrate from AlgE-1.9A on AlgE-2.8A illustrating the partial overlap in the positions of citrate and residues on T8 and L2 in the proposed alginate conduction pore. (*A*) Cutaway view of AlgE-2.8A is shown to reveal the channel interior. (*B*) Expanded view of the boxed area in (*A*).

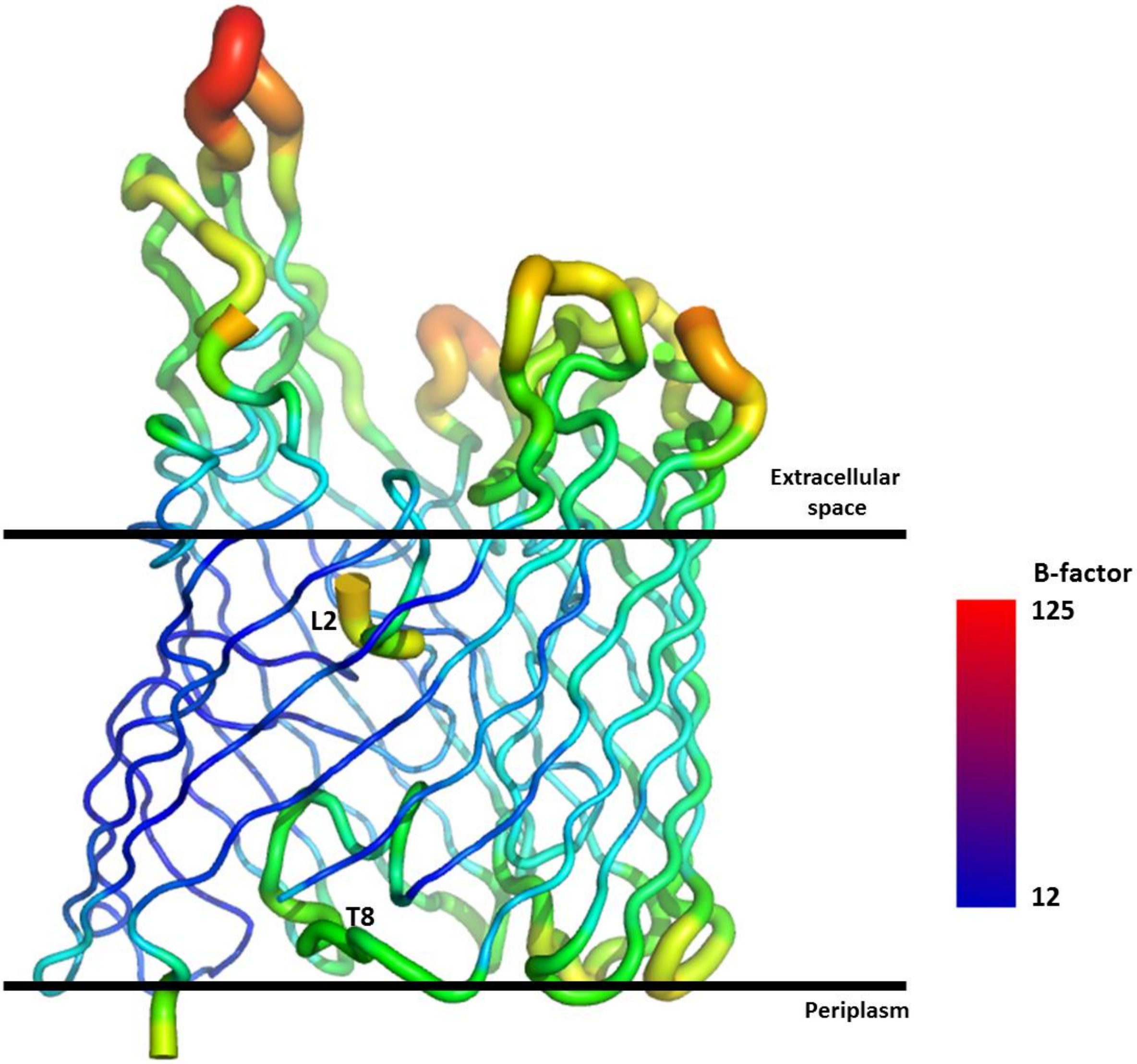

Figure S6 B-factor distribution within the AlgE structure shown in putty representation. AlgE-2.4B is shown and was chosen because of its completeness. The B-factor scale (Å^2), which runs from 12 (blue) to 125 (red), is shown on the right.

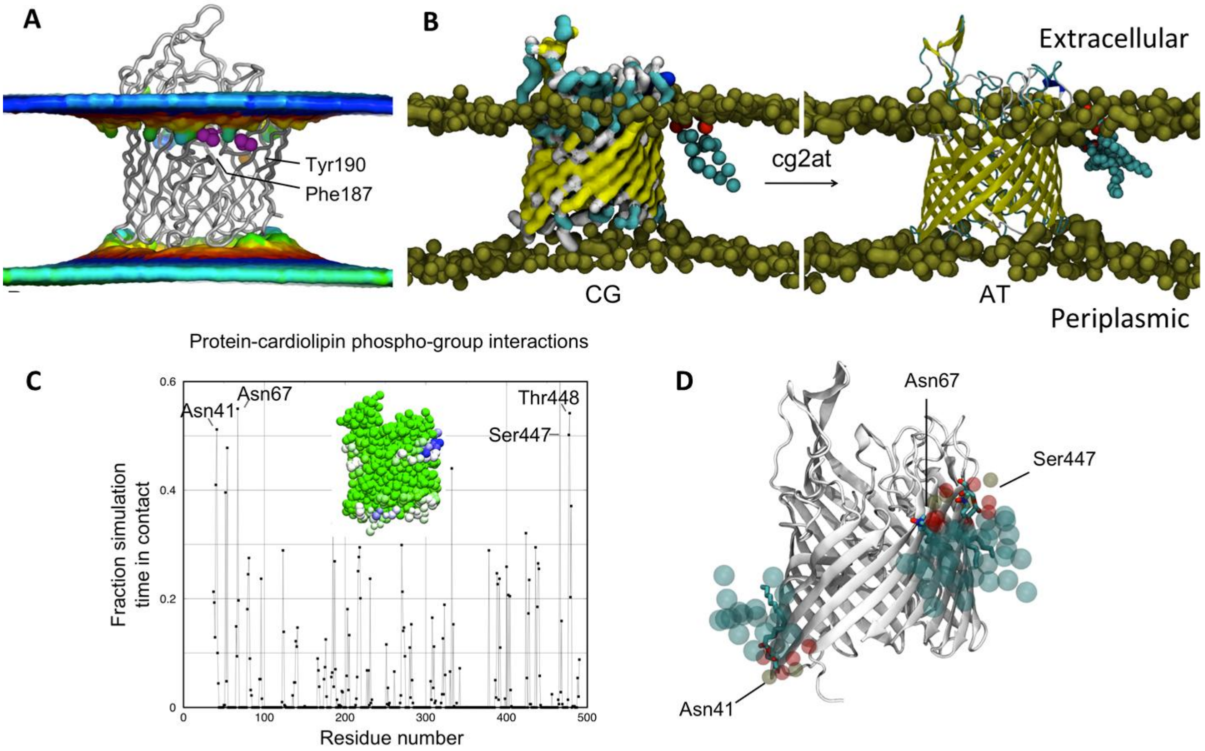

Figure S7 Interactions of AlgE with lipids. (*A*) Average positions of lipid phosphate particles over a 1 μs coarse-grained (CG) simulation shown as a surface. Surface is coloured in terms of deformation compared to the mean lipid head group height over the course of the simulation. Some thinning of the bilayer is observed in the region of the protein. Residues Phe187 and Tyr190 (shown in magenta space-filling representation) sit above the short S5 and S6 strands. These hydrophobic residues compensate for the short β-strands such that the membrane does not significantly deform in this region. (*B*) Final snapshot of a CG simulation in which the bilayer has self-assembled around the AlgE protein. This snapshot is converted to atomistic representation (AT) for further simulation. The protein is coloured by secondary structure (β-sheet, yellow; α-helix, blue; turn, cyan; coil, white). Lipid phosphate groups are shown as tan spheres. Lipid tails and water molecules are not shown. A bound cardiolipin molecule is shown in both CG and AT representations. (*C*) Residues in contact (defined as within a 6 Å cut-off) with cardiolipin head groups during the course of the 1 μs CGMD simulation. Three binding sites in which a cardiolipin molecule is bound for over half the simulation are indicated. The inset shows the protein as spheres coloured according to the average time in contact with a cardiolipin head group (green = 0 % through white up to blue = 55 %). (*D*) The three binding sites identified from analysis in (*C*) are shown here with CG lipids coloured as transparent spheres mapped on to the 4AFK crystal structure with resolved bound lipids shown in stick representation. We propose that the outer-leaflet binding sites would be occupied by LPS.

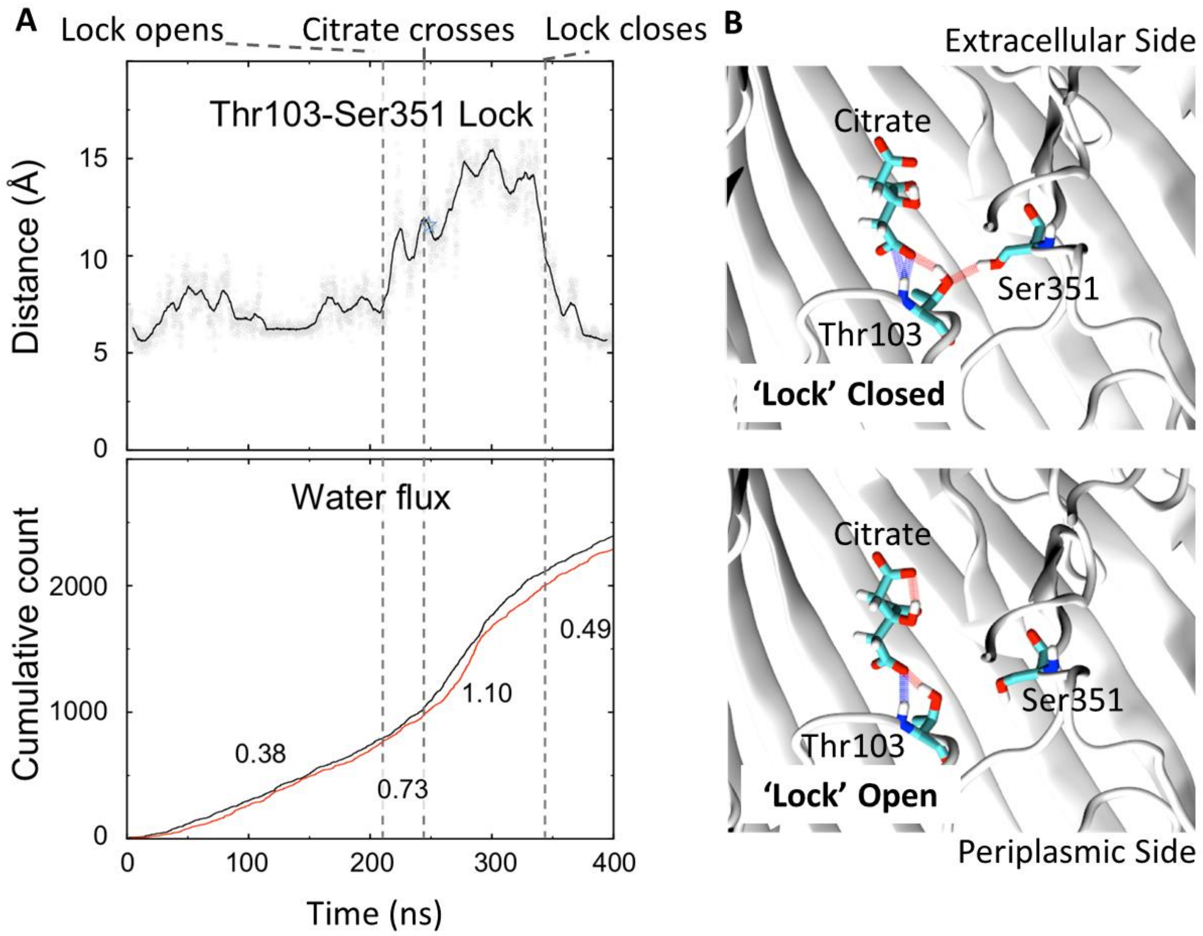

Figure S8 Water flux as a function of extracellular (E) gate opening. *(A)* The E gate opening may be measured in terms of the distance between residues Thr103 and Ser351 that form a hydrogen bonded 'lock' when the E gate is not fully opened (upper panel). The number of water molecules crossing the pore over the simulation is shown as a cumulative count (lower panel). The gradients are shown when the lock is closed, when the lock is first broken, as the citrate moves through the constriction site and after the lock closes again. As citrate passes through the pore at time ~245 ns, coincident with L2 motion (increase in distance between Thr103 on L2 and Ser351 on L7) there is a sharp increase in the rate of water passing through. Citrate exits the pore in a hydrated state. As the L2 loop moves back towards its resting configuration (time 380 ns) the rate of water crossing reduces to close to the original level (0.40 by the final 10 ns of the trajectory, compared to 0.38 at the start). The black line corresponds to water molecules crossing the pore from the periplasmic side to the extracellular side, i.e., travelling in the direction of citrate. The red line corresponds to water molecules travelling in the reverse direction. *(B)* Snapshots corresponding to the lock closed (upper panel) and open (lower panel) states as indicated. The Thr103 and Ser351 side chains as well as the citrate molecule are shown in stick representation. Hydrogen bonds between these residues and citrate are shown as dashed lines.

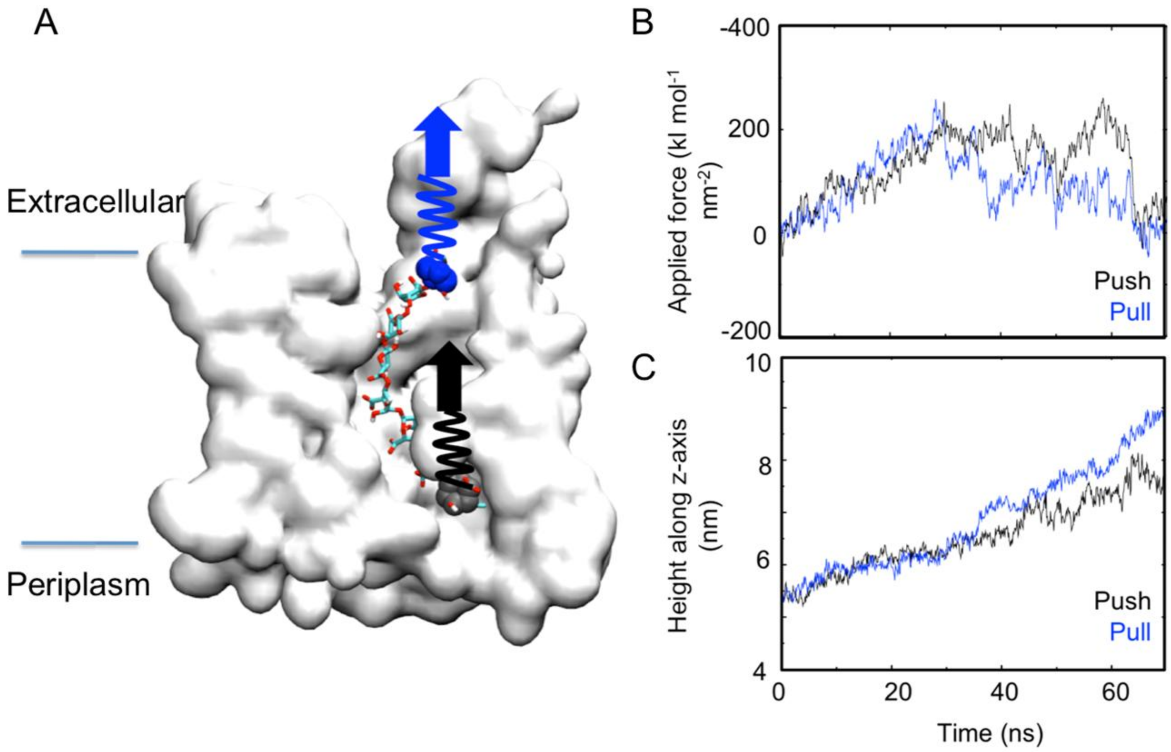

Figure S9 Steered molecular dynamics simulations of alginate being ‘pushed’ and ‘pulled’ through the AlgE pore. *(A)* The alginate is moved through the pore by attaching a harmonic spring to the terminal saccharides (coloured black for the push case and blue for the pulling case). The force is applied to the harmonic spring and directed along the z-axis in the direction from the extracellular side to the periplasmic side, indicated by arrows. *(B)* The force profiles (coloured as in *A*) give an indication of how much energy is required to move the alginate as it travels through the pore. The data corresponding to the push simulation is shown as In both cases the maximal applied force is similar (200 kJ mol^{-1} nm^{-2}). *(C)* The position along the pore of the sugar subunit proximal to the periplasmic side (coloured dark grey) is shown over time for both pushing and pulling simulations (coloured as in *A* and *B*). It is apparent from the non-linearity of these plots that the motion of alginate is not smooth, rather it moves through the pore in a step-like manner.

Table S1 Comparison of topology and model completeness between AlgE structures.

Region	AlgE-1.9 (4AFK)[a]		AlgE-2.4A (4B61)[a]		AlgE-2.4B (4B61)[a]		AlgE-2.8A (4AZL)[a]		AlgE-2.8B (4AZL)[a]		AlgE-2.3A (3BRH)		AlgE-2.3B (3BRH)		AlgE-2.3C (3BRH)		AlgE-2.3D (3BRH)	
	Res. / No.	Missing[b] Res. / No.	Res. / No.	Missing Res. / No.	Res. / No.	Missing Res. / No.	Res. / No.	Missing Res. / No.	Res. / No.	Missing Res. / No.	Res. / No.	Missing Res. / No.	Res. / No.	Missing Res. / No.	Res. / No.	Missing Res. / No.	Res. / No.	Missing Res. / No.
N-term.	33-41/9	33-36/4	33-41/9	33-38/6	33-41/9	33-37/5	33-41/9	33-39/7	33-41/9	33-38/6	33-41/9	33-39/7	33-41/9	33-39/7	33-41/9	33-39/7	33-41/9	33-39/7
Strand 1	42-54/13	-	42-54/13	-	42-54/13	-	42-54/13	-	42-54/13	-	42-54/13	-	42-54/13	-	42-54/13	-	42-54/13	-
Loop 1	55-65/11	-	55-65/11	-	55-65/11	-	55-65/11	63-64/2	55-65/11	63-64/2	55-65/11	-	55-65/11	-	55-65/11	-	55-65/11	-
Strand 2	66-81/16	-	66-81/16	-	66-81/16	-	66-81/16	-	66-81/16	-	66-81/16	-	66-81/16	-	66-81/16	-	66-81/16	-
Turn 1	82-83/2	-	82-83/2	-	82-83/2	-	82-83/2	-	82-83/2	-	82-83/2	-	82-83/2	-	82-83/2	-	82-83/2	-
Strand 3	84-95/12	-	84-95/12	-	84-95/12	-	84-95/12	-	84-95/12	-	84-95/12	-	84-95/12	-	84-95/12	-	84-95/12	-
Loop 2[c]	96-123/28	109-116/8	96-123/28	106-115/10	96-123/28	106-116/11	96-123/28	104-120/17	96-123/28	105-119/15	96-123/28	105-120/16	96-123/28	104-120/17	96-123/28	105-120/16	96-123/28	105-121/17
Strand 4	124-135/12	-	124-135/12	-	124-135/12	-	124-135/12	-	124-135/12	-	124-135/12	-	124-135/12	-	124-135/12	-	124-135/12	-
Turn 2[d]	136-143/8	-	136-143/8	-	136-**142**/7	-	136-143/8	-	136-143/8	-	136-143/8	-	136-143/8	-	136-143/8	-	136-143/8	-
Strand 5[d]	144-154/11	-	144-154/11	-	**143**-154/12	-	144-154/11	-	144-154/11	-	144-154/11	-	144-154/11	-	144-154/11	-	144-154/11	-
Loop 3	155-161/7	-	155-161/7	-	155-161/7	-	155-161/7	-	155-161/7	-	155-161/7	-	155-161/7	-	155-161/7	-	155-161/7	-
Strand 6	162-173/12	-	162-173/12	-	162-173/12	-	162-173/12	-	162-173/12	-	162-173/12	-	162-173/12	-	162-173/12	-	162-173/12	-
Turn 3	174-176/3	-	174-176/3	-	174-176/3	-	174-176/3	-	174-176/3	-	174-176/3	-	174-176/3	-	174-176/3	-	174-176/3	-
Strand 7	177-185/9	-	177-185/9	-	177-185/9	-	177-185/9	-	177-185/9	-	177-185/9	-	177-185/9	-	177-185/9	-	177-185/9	-
Loop 4[d]	186-203/18	-	186-**204**/19	-	186-**204**/19	-	186-203/18	-	186-203/18	-	186-203/18	-	186-203/18	-	186-203/18	-	186-203/18	-
Strand 8[d]	204-216/13	-	**205**-216/12	-	**205**-216/12	-	204-216/13	-	204-216/13	-	204-216/13	-	204-216/13	-	204-216/13	-	204-216/13	-
Turn 4	217-218/2	-	217-218/2	-	217-218/2	-	217-218/2	-	217-218/2	-	217-218/2	-	217-218/2	-	217-218/2	-	217-218/2	-
Strand 9[d]	219-230/12	-	219-**229**/11	-	219-**229**/11	-	219-230/12	-	219-230/12	-	219-230/12	-	219-230/12	-	219-230/12	-	219-230/12	-
Loop 5[d]	231-247/17	-	**230**-247/18	-	**230**-247/18	-	231-247/17	240-246/7	231-247/17	242-244/3	231-247/17	-	231-247/17	-	231-247/17	232-244/13	231-247/17	233-242/10
Strand 10	248-261/14	-	248-261/14	-	248-261/14	-	248-261/14	-	248-261/14	-	248-261/14	-	248-261/14	-	248-261/14	-	248-261/14	-
Turn 5	262-271/10	-	262-271/10	-	262-271/10	-	262-271/10	-	262-271/10	-	262-271/10	-	262-271/10	-	262-271/10	-	262-271/10	-
Strand 11[d]	272-292/21	-	272-**293**/22	-	272-**293**/22	-	272-**289**/18	-	272-**289**/18	-	272-**288**/17	-	272-**290**/19	-	272-**287**/16	-	272-**287**/16	-
Loop 6[d,e]	293-296/4	-	**294-295**/2	-	**294-295**/2	-	**290-300**/11	291-297/7	**290-300**/11	291-297/7	**289-301**/13	290-296/7	**291-298**/8	292-296/5	**288-303**/16	290-302/13	**288-303**/16	289-302/14
Strand 12[d]	297-319/23	-	**296**-319/24	-	**296**-319/24	-	**301**-319/19	-	**301**-319/19	-	**302**-319/18	-	**299**-319/21	-	**304**-319/16	-	**304**-319/16	-
Turn 6	320-323/4	-	320-323/4	-	320-323/4	-	320-323/4	-	320-323/4	-	320-323/4	-	320-323/4	-	320-323/4	-	320-323/4	-
Strand 13	324-333/10	-	324-333/10	-	324-333/10	-	324-333/10	-	324-333/10	-	324-333/10	-	324-333/10	-	324-333/10	-	324-333/10	-
Loop 7	334-376/43	-	334-376/43	-	334-376/43	-	334-376/43	-	334-376/43	-	334-376/43	-	334-376/43	-	334-376/43	-	334-376/43	-
Strand 14	377-388/12	-	377-388/12	-	377-388/12	-	377-388/12	-	377-388/12	-	377-388/12	-	377-388/12	-	377-388/12	-	377-388/12	-

Turn 7	389-391/3	-	389-391/3	-	389-391/3	-	389-391/3	-	389-391/3	-	389-391/3	-	389-391/3	-	389-391/3	-	389-391/3	-
Strand 15	392-403/12	-	392-403/12	-	392-403/12	-	392-403/12	-	392-403/12	-	392-403/12	-	392-403/12	-	392-403/12	-	392-403/12	-
Loop 8	404-424/21	-	404-424/21	-	404-424/21	-	404-424/21	-	404-424/21	-	404-424/21	-	404-424/21	-	404-424/21	-	404-424/21	-
Strand 16	425-437/13	-	425-437/13	-	425-437/13	-	425-437/13	-	425-437/13	-	425-437/13	-	425-437/13	-	425-437/13	-	425-437/13	-
Turn 8 [c]	438-456/19	439-454/16	438-456/19	441-453/13	438-456/19	-	438-456/19	-	438-456/19	-	438-456/19	-	438-456/19	-	438-456/19	-	438-456/19	-
Strand 17	457-467/11	-	457-467/11	-	457-467/11	-	457-467/11	-	457-467/11	-	457-467/11	-	457-467/11	-	457-467/11	-	457-467/11	-
Loop 9	468-478/11	-	468-478/11	-	468-478/11	472-474/3	468-478/11	-	468-478/11	-	468-478/11	-	468-478/11	-	468-478/11	-	468-478/11	-
Strand 18	479-489/11	-	479-489/11	-	479-489/11	-	479-489/11	-	479-489/11	-	479-489/11	-	479-489/11	-	479-489/11	-	479-489/11	-
C-term.	490/1	-	490/1	-	490/1	-	490/1	-	490/1	-	490/1	-	490/1	-	490/1	-	490/1	

[a] Structure solved as part of this work. [b] Refers to residues that lack electron density. [c] Loop 2 and Turn 8, the two regions where most of the discussion is focused, are underlined. [d] Regions that vary in topology between the different models. Differences with AlgE-1.9 are highlighted in bold. [e] Loop and strand designations are based on the AlgE-1.9 model.

References

Caffrey, M., D. Li & A. Dukkipati (2012). *Biochemistry*, **51**, 6266-6288.

Lomize, M. A., I. D. Pogozheva, H. Joo, H. I. Mosberg & A. L. Lomize (2012). *Nucleic Acids Res.*, **40**, D370-D376.

Whitney, J. C., I. D. Hay, C. H. Li, P. D. W. Eckford, H. Robinson, M. F. Amaya, L. F. Wood, D. E. Ohman, C. E. Bear, B. H. Rehm & P. L. Howell (2011). *Proc. Natl. Acad. Sci. U.S.A.*, **108**, 13083-13088.
