# Supplementary material for: A conformational landscape for alginate secretion across the outer membrane of Pseudomonas aeruginosa
Source: Acta Crystallogr D Biol Crystallogr. 2014 Jul 25;70(Pt 8):2054–68. doi: 10.1107/S1399004714001850 (PMC4118822; doi:10.1107/S1399004714001850)
Supplement: Supplementary file 3 [file d-70-02054-sup4.pdf]

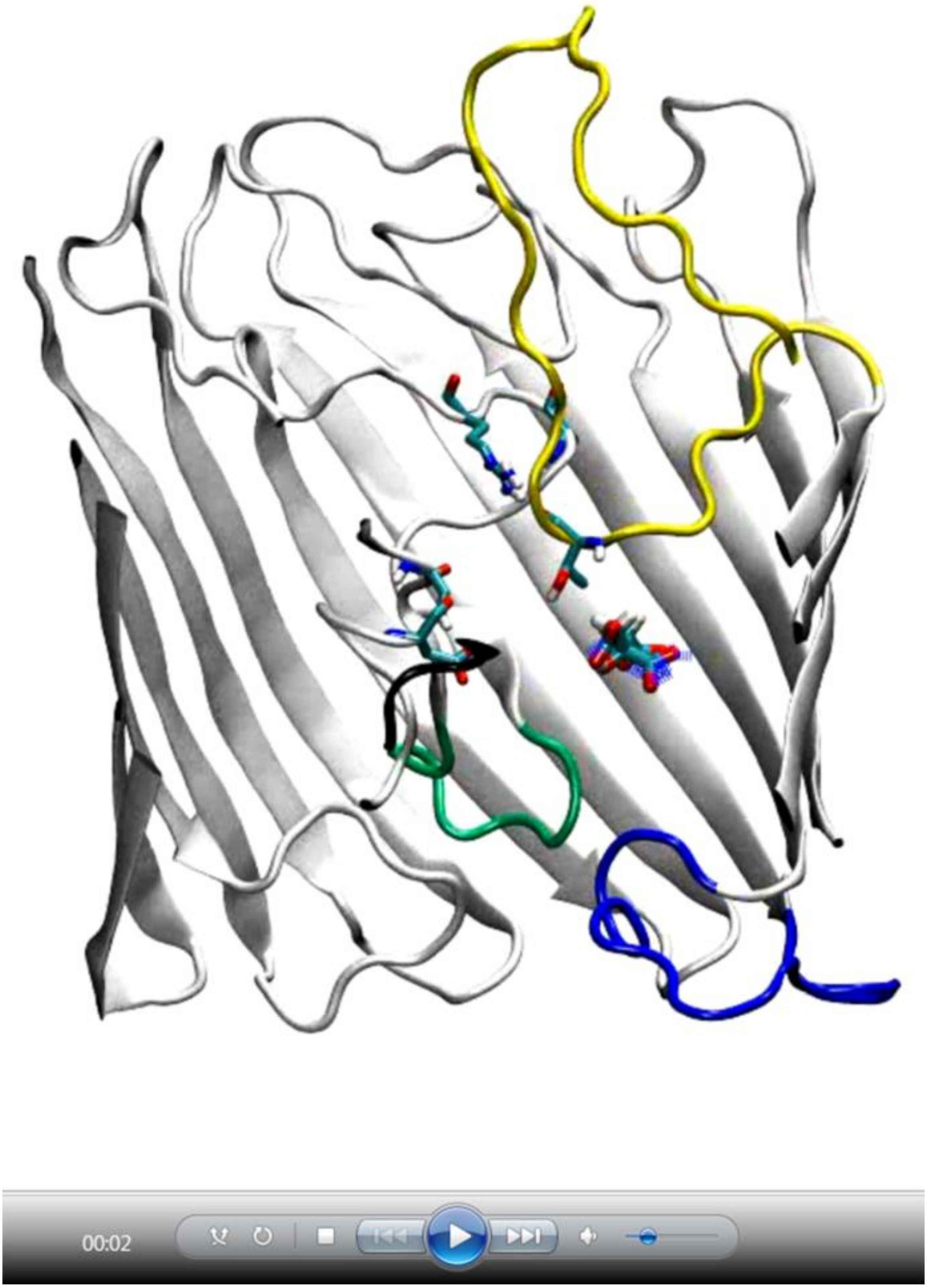

Movie 1. Citrate exit through AlgE pore as shown in Fig 4. The entire 400 ns trajectory is shown. Colouring and representations are as described in the legend to Fig 4.
