# Supplementary material for: A conformational landscape for alginate secretion across the outer membrane of Pseudomonas aeruginosa
Source: Acta Crystallogr D Biol Crystallogr. 2014 Jul 25;70(Pt 8):2054–68. doi: 10.1107/S1399004714001850 (PMC4118822; doi:10.1107/S1399004714001850)
Supplement: Supplementary file 5 [file d-70-02054-sup6.pdf]

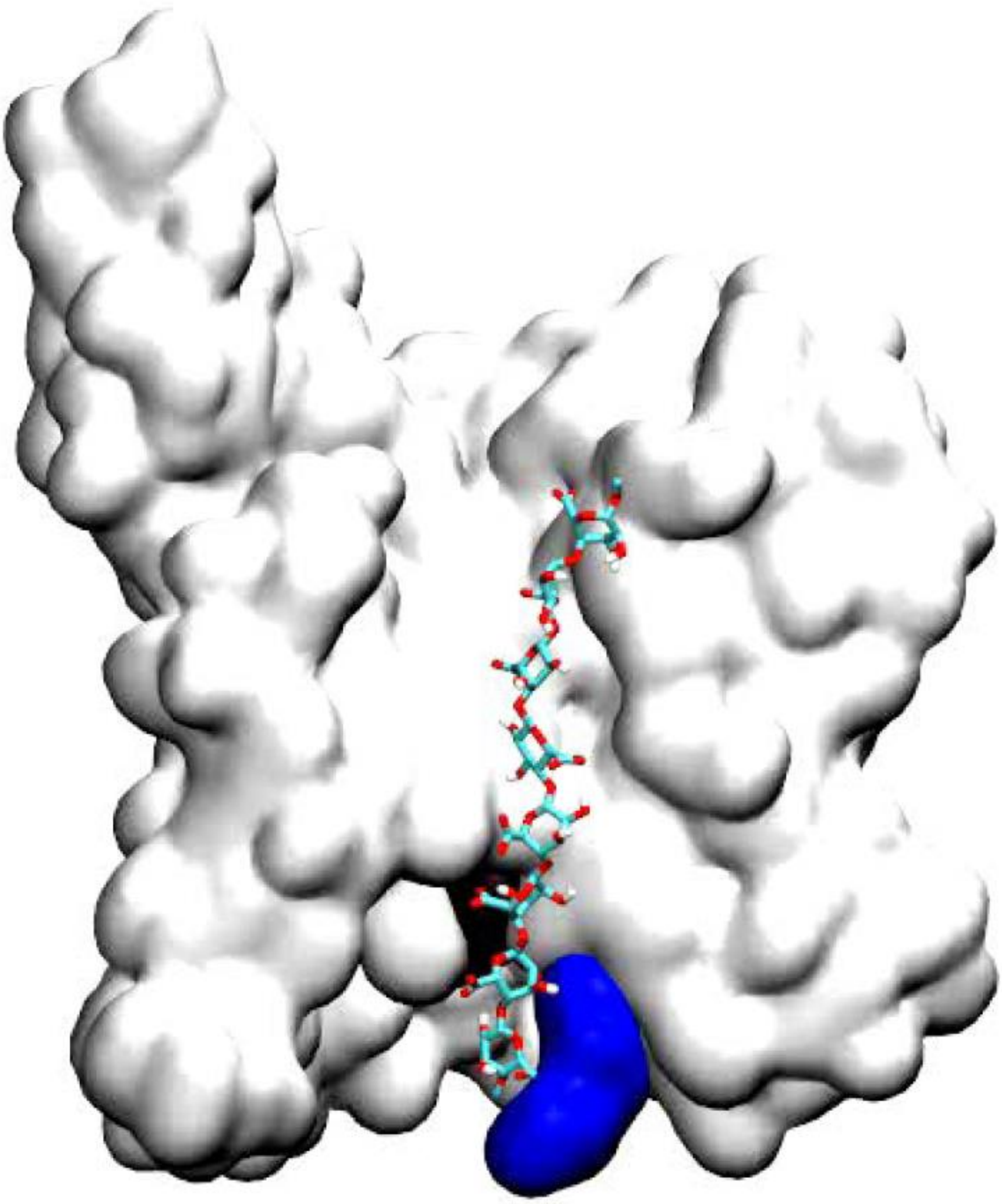

Movie 2. Steered molecular dynamics simulation of alginate octamer being ‘pushed’ through the pore of AlgE. This movie corresponds to the pushing simulation described in Fig. S6. This movie shows the same 100 ns trajectory twice from different views (in the second half the view point is rotated 180 ° around the membrane normal). The protein is shown as a surface. A slice view of the protein, cutaway in one direction of the membrane plane, allows the alginate molecule to be observed. Alginate is displayed in stick representation. In this simulation a force is applied to the bottom sugar unit of the alginate polymer to ‘push’ it upwards through the pore to the extracellular side of the protein. The motion of the alginate is not smooth, corresponding to the plots in Fig S6. The pore of the protein is largely immobile whilst the alginate moves through. The T8 (blue), L3 (black) and L2 (yellow) loops are coloured individually.
